# Spatially varying cis-regulatory divergence in *Drosophila* embryos elucidates cis-regulatory logic

**DOI:** 10.1371/journal.pgen.1007631

**Published:** 2018-11-01

**Authors:** Peter A. Combs, Hunter B. Fraser

**Affiliations:** Department of Biology, Stanford University, Stanford, California, United States of America; New York University, UNITED STATES

## Abstract

Spatial patterning of gene expression is a key process in development, yet how it evolves is still poorly understood. Both cis- and trans-acting changes could participate in complex interactions, so to isolate the cis-regulatory component of patterning evolution, we measured allele-specific spatial gene expression patterns in *D. melanogaster* × *simulans* hybrid embryos. RNA-seq of cryo-sectioned slices revealed 66 genes with strong spatially varying allele-specific expression. We found that *hunchback*, a major regulator of developmental patterning, had reduced expression of the *D. simulans* allele specifically in the anterior tip of hybrid embryos. Mathematical modeling of *hunchback* cis-regulation suggested a candidate transcription factor binding site variant, which we verified as causal using CRISPR-Cas9 genome editing. In sum, even comparing morphologically near-identical species we identified surprisingly extensive spatial variation in gene expression, suggesting not only that development is robust to many such changes, but also that natural selection may have ample raw material for evolving new body plans via changes in spatial patterning.

## Author summary

Connecting changes in gene regulatory sequences to changes in expression remains an open problem in biology. Measuring allele-specific expression in interspecific F1 hybrids is a powerful way to identify genes with differential cis-regulation, since both parental copies are exposed to the same trans-regulatory environment in a given sample. To identify genes with different regulation during embryonic development between the closely related fruit fly species *D. melanogaster* and *D. simulans*, we sliced embryos along their anterior-posterior axis and identified dozens of genes with spatial differences in their regulation. We used mathematical modeling to identify likely regulatory changes in one of these genes, *hunchback*, and we found a single candidate variant that, upon testing, did indeed have the predicted effect on *hunchback’s* expression. This work presents a new, integrated approach for probing regulatory logic by sensitively searching for cis-regulatory differences followed by tightly directed predictions of causal changes.

## Introduction

Although most cells in any metazoan share the same genome, they nevertheless diversify into an impressive variety of precisely localized cell types during development. This complex spatial patterning is due to the precise expression of genes at different locations and times during development. Where and when each gene is expressed is largely dictated by the activities of cis-regulatory modules (CRMs, which include enhancers, insulators, and other regulatory elements) through the binding of transcription factors to their recognition sequences [1–3]. Despite the importance of these patterning CRMs for proper organismal development, they are able to tolerate some modest variation in sequence and level of activity [4–7]. Indeed, this variation is one of the substrates upon which selection can act. However, even in the handful of cases where we understand the regulatory logic, efforts to predict the effects of inter-specific differences in CRMs still have limited precision [8–10].

A complicating factor in comparing gene expression between species is that both cis- and trans-acting regulation in a given cell type can change [11]. Furthermore, subtle differences in embryo size across *Drosophila* species means that nuclei from the same spatial position may not be of an identical cell type [12, 13]. A solution to both of these issues is to focus on cis-regulatory changes by measuring allele-specific expression (ASE) in F1 hybrids. In a hybrid each diploid nucleus has one copy of each parent’s genome which is exposed to the same set of trans-regulatory factors, so any differences in zygotic usage of the two copies is due either to cis-regulatory divergence or to stochastic bursting (pulses of transcription due to the independent release of polymerase which should be averaged out over many cells) [14]. In addition, even if cell positions between the two parental species have shifted, focusing on a hybrid will sidestep this complication since the alleles in any subset of cells are derived from exactly the same hybrid cells.

The early *Drosophila* embryo provides a unique opportunity to probe the interaction of trans-regulatory environments with cis-regulatory sequence: by slicing the embryo along the anterior-posterior axis, we are able to measure ASE in nuclei that are physically close, and therefore that have similar complements of transcription factors (TFs). We reasoned that by combining knowledge of the regulatory sequence changes between the species with the transcription factors expressed in each slice, it should be possible to more quickly identify which genetic variant(s) underlie the expression difference.

In this study, we used spatially-resolved transcriptome profiling to search for genes where cis-regulatory differences drive allele-specific expression patterns in hybrid *D. melanogaster* × *D. simulans* embryos (specifically the reference strains DGRP line 340 for *D. melanogaster* and *w*^501^ for *D. simulans*; unless otherwise noted, we will refer to the two reference strains, and not the two species as a whole). We found dozens of genes with clear, consistent differences in allele-specific expression across the embryo. We chose one of these genes, *hunchback (hb)*, as a case study. Mathematical modeling of *hunchback* cis-regulation suggested that the gain of a weak binding site for Bicoid and Huckebein was responsible for much of the expression difference, which we confirmed through CRISPR-Cas9 mediated editing of the endogenous *D. melanogaster* locus.

## Results

### A genome-wide atlas of spatial gene expression in *D. melanogaster* × *D. simulans* hybrids

**Fig 1:**
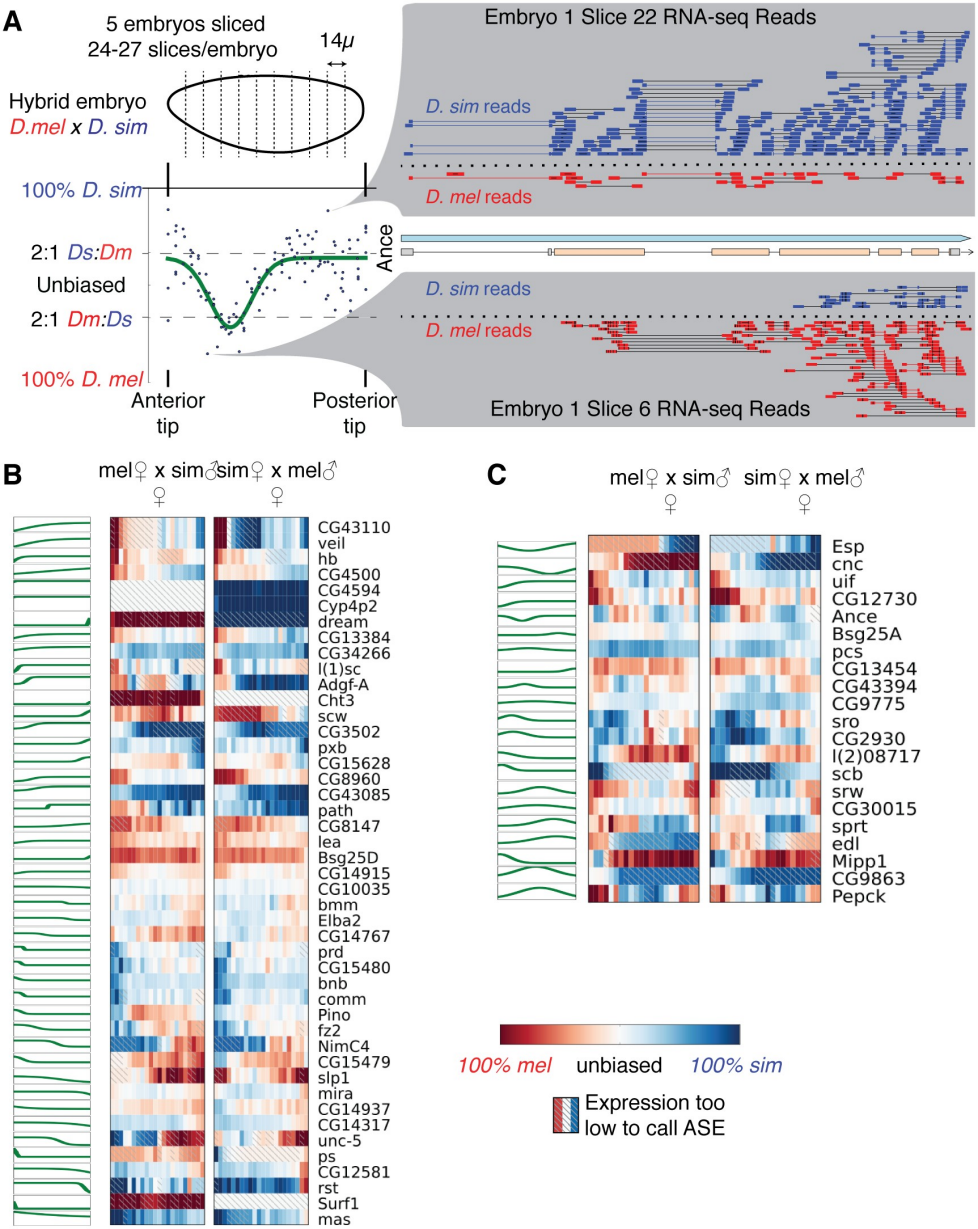
RNA-seq of hybrid *Drosophila* embryos reveals extensive spatially patterned allele-specific expression. A) Each embryo was cryosliced along the anterior-posterior axis in 14μm sections, followed by RNA-seq in each slice. Allele-specific expression (ASE) was called for each gene in each slice by assigning unambiguous reads to the parent of origin; shown here are the reads for the gene *Ance*, with blue indicating *D. simulans* reads and red indicating *D. melanogaster* reads. For each gene, we fit either a step-like or peak-like (shown) function. B-C) Genes with a step-like pattern (B, best fit by a logistic function) or peak-like pattern (C, best fit by a Gaussian function). For each gene, anterior is left and posterior is right. The green line indicates the best fit pattern, with higher indicating *D. simulans* biased expression, and lower indicating *D. melanogaster* biased expression. The heat maps are from the first female replicate of each direction of the cross.

We selected five hybrid embryos at mid-stage 5, with membrane invagination between 50 and 65% (approximately 150 minutes after fertilization). We then sliced the embryos to a resolution of 14μm, yielding between 25 and 27 slices per embryo (Fig 1A). We chose embryos from reciprocal crosses (i.e. with either a *D. melanogaster* mother or a *D. simulans* mother), and had at least one embryo of each sex from each direction of the cross. Although hybrid female embryos derived from a *D. simulans* mother are typically embryonic lethal at approximately this stage [15], the *w*^501^ strain we used is an exception to this and both sexes of its hybrid progeny develop normally [16]. We also sliced one embryo from each of the parental strains. Following slicing, we amplified and sequenced poly-adenylated mRNA using SMART-seq2 with minor modifications (see Methods) [17–19]. To assess the quality and reproducibility of our RNA-seq data, we compared expression levels between spatially matched slices from different embryos, and found strong concordance (r = 0.973 ± 0.008; S1 Fig).

We first searched for cases of hybrid mis-expression—genes where the absolute expression pattern is consistently different in the hybrid, compared to the parents alone. Using earth-mover distance (EMD) to measure differences in expression patterns (S2 Fig; [20]), for each zygotically expressed gene we compared the expression pattern from each of the hybrid embryos to the pattern expected by taking the average of the *D. melanogaster* and *D. simulans* embryos. After Benjamini-Hochberg FDR correction, no gene was significantly more different from the average of the parental embryos than each of the parental embryos were from each other (smallest q-value = .37, see Methods). We also compared expression patterns of hybrid embryos with a *D. melanogaster* mother to those with a *D. simulans* mother, and found that most differences seemed to be due to differing patterns of maternal deposition or noisy expression (S3 Fig). Thus, we conclude that there do not seem to be any expression patterns that are not explained by differences in the parents or that are unique to the hybrid context.

### Spatially varying allele-specific expression highlights genes with cis-regulatory changes

Comparisons of patterns in absolute expression data suffer from difficulties in comparing embryos of subtly different sizes and stages, especially when those embryos are of different species. However, this is not a concern for genes with spatially varying allele-specific expression (svASE)—that is, expression in one part of the embryo that is differently biased compared to another part of the same embryo. Statistical tools for identifying spatial patterns in continuously varying data are limited compared to more traditional treatment/control designs [21, 22]. Therefore, we chose to use a simple ASE score, the ratio of the difference between the number of *D. simulans* and *D. melanogaster* reads and the sum of the number of reads,

$$\begin{array}{c} ASE=\frac{n_{sim}-n_{mel}}{n_{sim}+n_{mel}} \end{array}$$ (1)

which is robust to the depth of sequencing of each sample and bounded between -1 (100% *D. melanogaster*) and 1 (100% *D. simulans*). These properties also facilitate mathematical fitting of svASE across samples.

As expected, we found that overall levels of ASE were significantly higher in genes that had been previously classified as maternally deposited in a developmental time course [23], while zygotic genes had much lower levels of ASE (S5 Fig). Aside from these maternally deposited genes, and consistent with previous observations at other stages [24, 25], we did not find any convincing evidence of imprinting (i.e. zygotic transcription of the paternal copy of a gene).

In order to identify genes with svASE, we fit two different simple patterns to the ASE as a function of embryo position (Fig 1A). Manual inspection of the data suggested that there were two primary patterns that appeared in the data: some genes had ASE biased towards one species at one extreme of the embryo then gradually transitioned to a different level of bias at the other end, while other genes had an approximately constant baseline of ASE with a relatively confined region that had a different level of ASE. We found that fitting a step-like (logistic) function did a reasonable job of identifying the first class, while a peak-like (Gaussian) function fit the second. While more complicated ASE patterns are conceivable, we did not observe any genes with such patterns.

We identified 45 genes where a step-like function explained at least 45% of the variance in ASE (Fig 1B and S6 Fig), and 21 where a Gaussian function explained at least that much of the variance (Fig 1C and S6 Fig; if both explained over 45% of the variance for a gene, we only count the one that better explains the variance). In order to estimate a false discovery rate, we shuffled the *x*-coordinates of the ASE values, and refit the functions. Of 1000 shuffles, only 6 (sigmoid) and 0 (peak) genes cleared the threshold for svASE, which implies false discovery rates of ≈0.020396% (sigmoid) and <0.001925% (peak). We selected the 45% variance-explained cutoff manually as a point where the patterns are visually clear; at a more relaxed 10% FDR cutoff, we found 320 genes where fitting explains at least 12% of the variance in ASE.

We were curious whether genes with svASE were enriched for any Gene Ontology (GO) terms that might indicate selection on a particular function or pathway [26, 27]. We found enrichments for genes involved in “embryonic morphogenesis” (GO:0048598, q-value 2.3 × 10^−6^), including “transcription factors” (GO:0003700, q-value 9.8 × 10^−7^) and “transmembrane receptors” (GO:0099600, q-value 2.2 × 10^−2^). These included key components in important signaling pathways, such as *fz2* (a Wnt receptor) and *sog* (a repressor of the TGFβ—signaling pathway). *Myc*, a cell cycle regulator that is a target of both of these pathways, also had significant svASE. However, when we used all non-uniformly expressed genes from [28] as a background set, we did not find any enriched GO terms, suggesting that the enrichments are driven by functions shared by spatially patterned genes overall, rather than among svASE genes specifically.

Finally, we looked for genes that are consistently biased towards one species, regardless of parent and spatial pattern. We found 84 genes with strongly *D. melanogaster*-biased ASE, and 39 genes with strongly *D. simulans*-biased ASE (S7 Fig). Given that the gene models we are using are taken entirely from *D. melanogaster*, we may be underestimating the true quantity of *D. simulans* biased genes (this caveat does not apply to spatially varying ASE, since inaccurate gene models would not lead to spatial variation across the embryo). Intriguingly, a few of these genes are expressed at comparable levels and with similar spatial patterns in the *D. melanogaster* and *D. simulans* parental embryos, indicating they may be affected by compensatory cis- and trans-acting changes. These species-biased genes are spread throughout the genome, suggesting that this effect is not a consequence of a single cis-regulatory change or inactivation of a single chromosomal region.

### A single SNP contributes to svASE in the gap gene *hunchback*

We noticed that *hunchback (hb)*, an important transcriptional regulator [8, 29, 30], had strong svASE (step-like fit *r*^2^ = 0.57; Fig 1B). Since the regulation of *hb* is relatively well-characterized, this provided the opportunity to study the sequence-level causes of the svASE that we observed.

**Fig 2:**
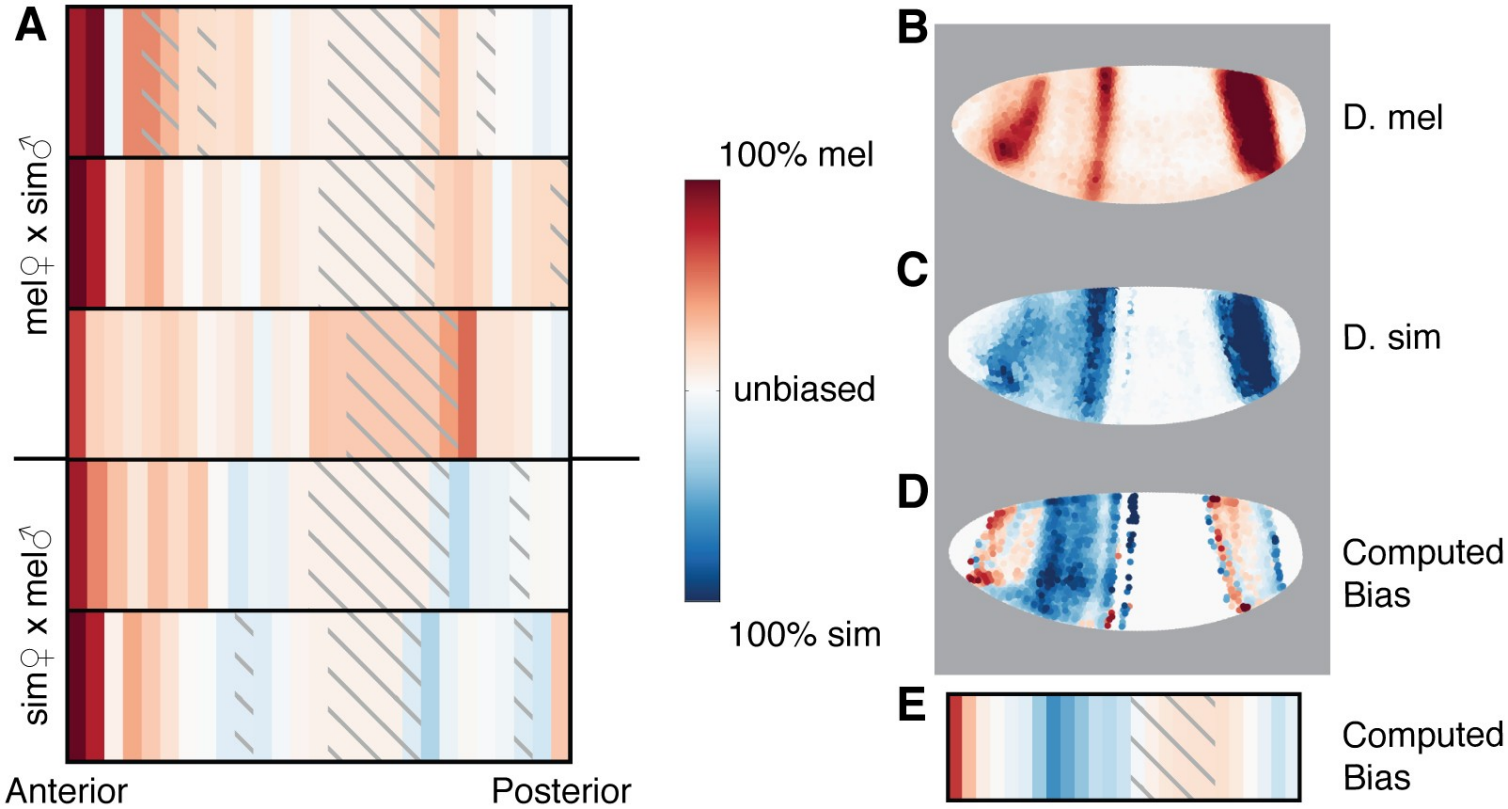
Hybrid embryos show strong melanogaster-specific expression of *hunchback* in the anterior. A) Heat map of svASE of *hb* shows a significant *D. melanogaster* bias in the anterior tip of the embryo. Each row is a different embryo. Embryos with a melanogaster mother are above the horizontal line. B-C) *In situ* hybridization for *hb* in parental embryos at the 26-50% membrane invagination stage from [31] (C) and [13] (D). Images are arranged anterior to the left and dorsal up. D) Computed bias for each nucleus. Nuclei with low expression in both species (less than 20% of the peak expression value) are colored white to reflect no callable bias. E) Overall computed bias for each 4% section of the embryo by x-position. *D. melanogaster* and *D. simulans* expression levels are summed for each nucleus in that section of the embryo, then bias computed. Bias is not computed for the middle sections of the embryo where no RNA-seq bias data is available due to low *hb* expression.

The *hb* svASE was driven by the anterior tip, which had a strong bias towards the *D. melanogaster* allele (Fig 2A). Although the anterior tip has lower expression levels of hunchback than other parts of the embryo, there were more than enough reads to be confident that there is a bias (between 38 and 599 assignable reads per library in the anterior-most slice). Compared to ASE elsewhere in the embryo, ASE in the anterior tip was both stronger (∼10-fold more *D. melanogaster* transcripts than *D. simulans*), and also less affected by the species of the mother (excluding the first six anterior slices, there are 5-15% more reads from the maternal species than the paternal). When we compared expression in two quantitative atlases of *hb* expression [13, 31], we found that although the *D. melanogaster* and *D. simulans* expression patterns were qualitatively quite similar (Fig 2B and 2C), a more careful analysis of the *hb* expression revealed more expression in nuclei at the anterior tip of *D. melanogaster* embryos than in matching nuclei of *D. simulans* (Fig 2D and S8 Fig). Computing expected allele-specific bias (see Methods) in each slice of the embryo showed strong qualitative and quantitative agreement with the actual ASE (Fig 2E; Pearson *r* = 0.62).

**Fig 3:**
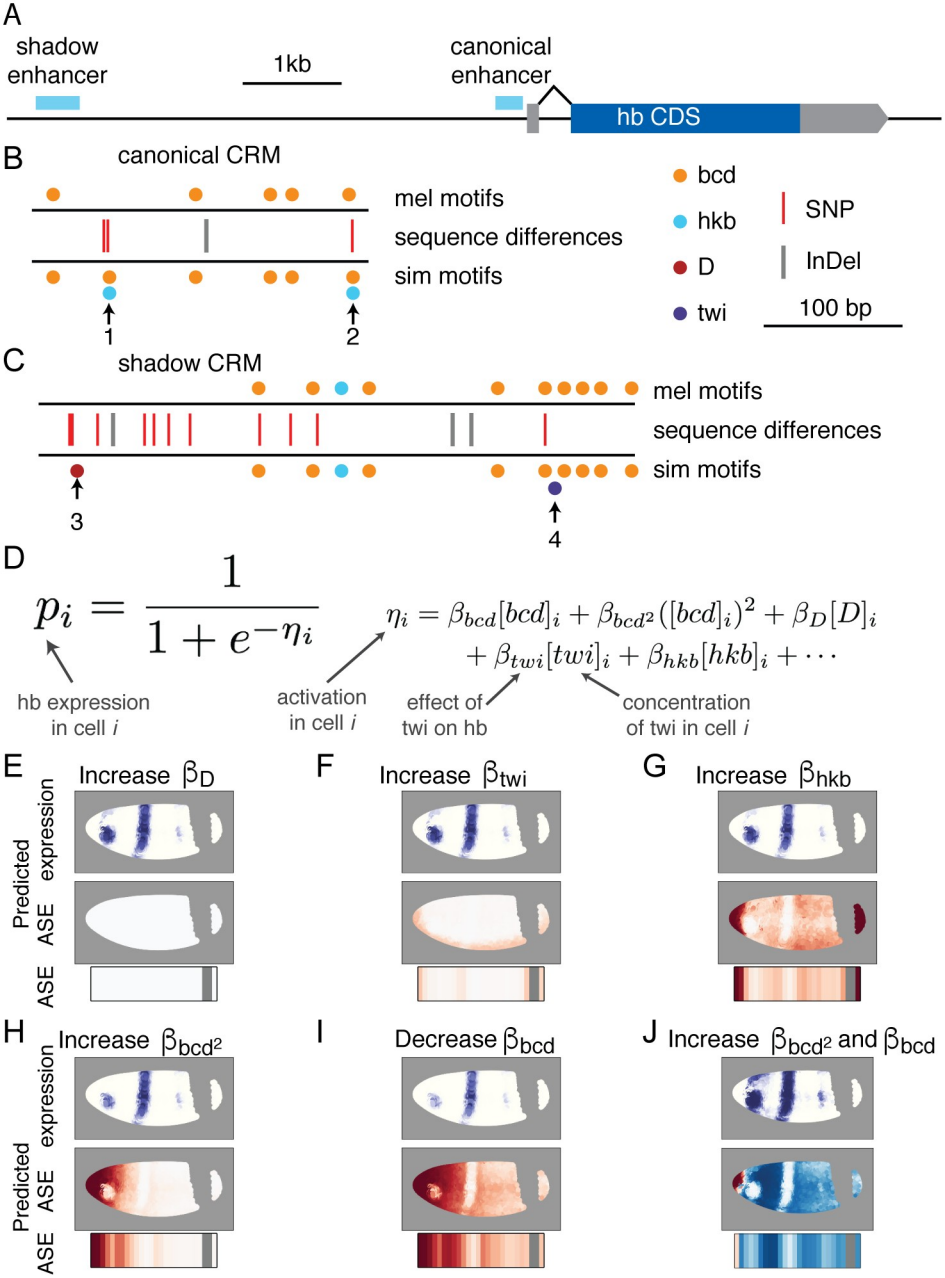
Cis-regulatory changes in *hb* regulatory regions could cause the observed svASE. A) Regulatory elements near the zygotic *hunchback-RA* transcript. B-C) FIMO binding motifs and inter-specific variants of the anterior activator (B) and shadow CRM from [35] (C). Species-specific predicted binding sites are highlighted with arrows. D) Overview of the logistic expression model. A function is fit for wild-type *D. melanogaster*, then individual activation/repression coefficients are independently adjusted for each TF. E-J) Predicted ASE from adjusting strength of each TF in the model in order to maximize the variance in the real ASE explained by the predicted ASE. Predicted absolute expression is shown in purple above, ASE per nucleus is shown in the middle panel in opposed red/blue, and predicted ASE in a sliced embryo is shown below. Note that in panel I, although a Bicoid site is gained in *D. simulans*, the best fit according to the model would be to decrease the coefficient.

We next examined known regulatory sequences near *hb* for changes in TF binding sites that might cause the strong ASE in the anterior tip of the embryo. We downloaded from RedFly all known CRMs and reporter constructs with *hb* as a target [32]. There are three known minimal CRMs for *hb* that have been tested for embryonic activity using transgenic constructs: the canonical anterior CRM proximal to the *hb* P2 promoter [33, 34], a more distal “shadow” CRM [35], and an upstream CRM that drives expression in both the anterior and posterior domains, but not the anterior tip of *D. melanogaster* [36] (Fig 3A). We excluded the upstream CRM from further consideration and used FIMO to scan the other regulatory sequences for motifs of the 14 TFs with ChIP signal near *hb* [37, 38]. Binding in the canonical Bicoid-dependent anterior element gained a single weak Bicoid motif and two very weak Huckebein (Hkb) motifs in *D. simulans* relative to *D. melanogaster* (Fig 3B, S9 Fig), with one of the gained Hkb motifs overlapping the gained Bcd motif. The distal “shadow” CRM gained twist and Dichaete binding motifs between *D. melanogaster* and *D. simulans* [3, 35] (Fig 3C). Unsurprisingly, binding sites for other TFs outside the core regulatory elements displayed pervasive apparent turnover, with multiple gains and losses between the species (S9 Fig) [5, 39].

Anterior zygotic expression of *hb* is driven primarily by Bicoid, but there are details of the expression pattern at mid-stage 5 that cannot be explained by the relatively simple Bicoid gradient, and the loss of expression at the anterior tip of *D. simulans* cannot be explained by additional Bicoid-dependent activation. In order to more fully understand how this pattern might be specified and what the effects of binding site changes could be, we took a modeling-based approach similar to [40]. We used the 3-dimensional gene expression atlas from [31] to test regulators in a logistic model for the anterior *hunchback* expression domain (see Methods). The model included a linear term for every gap gene TF bound in the anterior activator CRM [37] and a quadratic term for Bicoid to account for observations that it may lose its ability to act as an activator at high concentrations [40, 41]. The best fit model (S2 Table, S10 Fig) had the strongest coefficients for the two Bicoid terms, consistent with previous studies examining *hb* output as a simple function of Bcd concentration [3, 33, 42]. The other TFs that bind to the locus are understood to be either repressors or have unclear direction of effect, and the coefficients for those TFs are negative or only weakly positive [8, 43, 44]. The exceptions to this are D and twi which act as weak activators in the model, and may be related to observations in the literature of bifunctionality for these TFs [45, 46].

We built this model to determine whether any of the binding site changes between *D. melanogaster* and *D. simulans* could plausibly explain the ASE that we observe in *hb*. Therefore, we did not make any effort to determine the minimal set of TFs that would drive the *hb* pattern, nor did we include a term to model predicted auto-regulation [47, 48]. Furthermore, the model underestimates the quantitative expression levels outside of the strongest part of the anterior stripe. As a result, it may be both overfit and an imperfect representation of the underlying cis-regulation of *hb* transcription, but should nevertheless indicate the likely effects of changes in TF binding.

In order to predict what effect the TF binding changes would have on expression in a *D. simulans* (or hybrid) embryo, we adjusted the coefficient for each TF independently to find the coefficient that best predicted the observed ASE. We then compared the output of the *D. melanogaster* model to the adjusted one (Fig 3E–3J). Adjusting the Bcd coefficients, either alone or in tandem (Fig 3H–3J), and increasing the Hkb coefficient (Fig 3G) produced a predicted ASE pattern quite similar to the actual expression differences we observed between the species. Furthermore, adjusting the coefficients for the TFs with binding changes in the shadow CRM did not yield strong correlation with the observed ASE. We therefore hypothesized that the combined Bcd/Hkb site is involved in the lower expression of the *D. simulans* *hb* anterior domain. Because the FIMO binding score for Hkb was very weak and its predicted presence depended on the specific Hkb position weight matrix used, we decided to focus on the combined Bcd/Hkb (Fig 3B site 1) rather than the Hkb-only locus (site 2).

**Fig 4:**
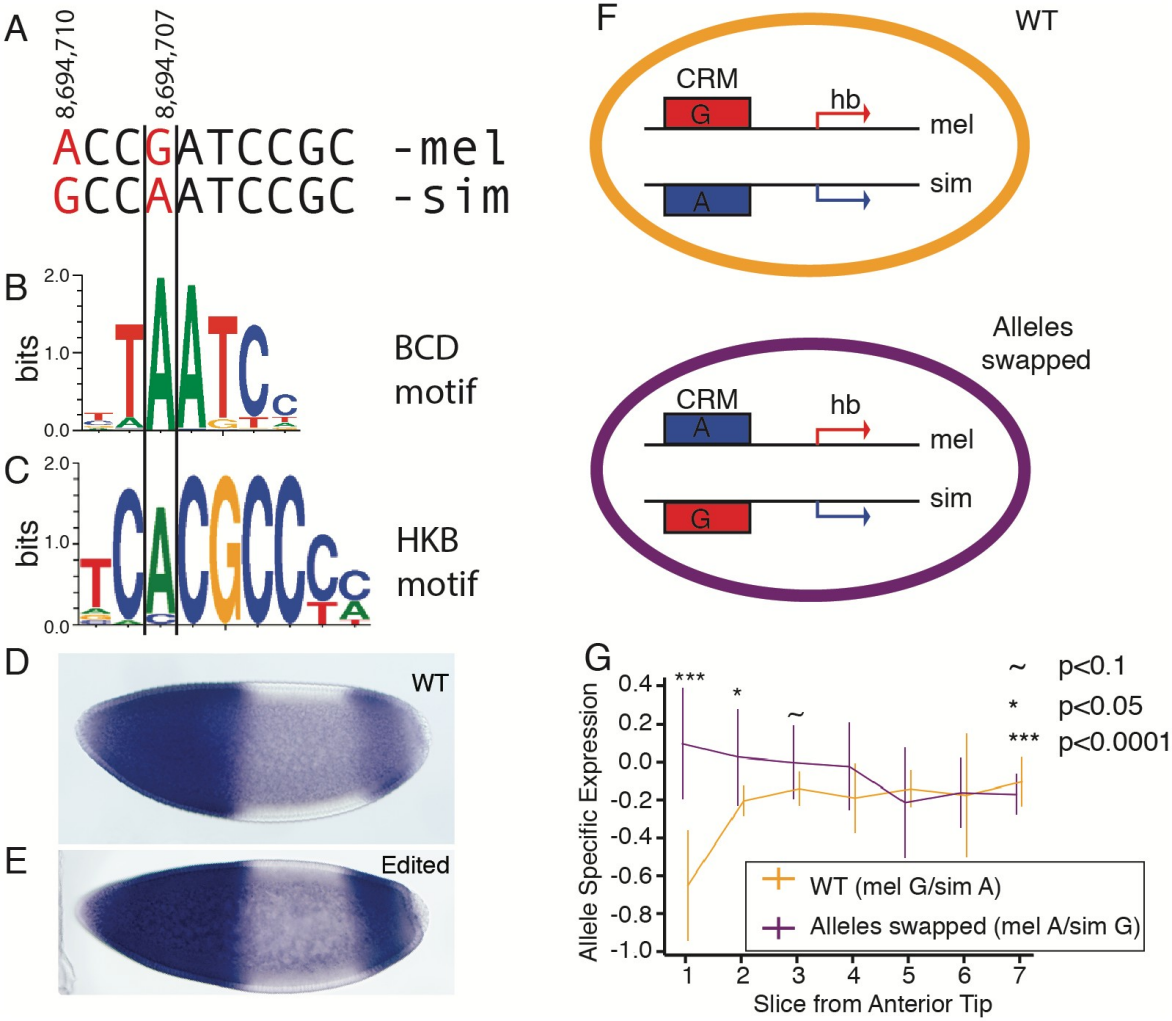
CRISPR-Cas9-mediated editing shows a Bicoid site in *D. simulans* is responsible for the change in expression pattern. A) A pair of SNPs in the canonical *hb* CRM at the indicated coordinates on *D. melanogaster* chromosome 3R. SNPs between *D. melanogaster* and *D. simulans* marked in red. B) The Bicoid binding motif aligned to the site of the binding change. C) The Huckebein binding motif aligned to the site of the binding change. D-E) Staining of *hb* in the two most closely staged wild-type (D) and CRISPR-edited (E) *D. melanogaster* embryos. F) We created hybrid embryos with the wild-type alleles on the wild-type chromosomes (orange), or with the *D. simulans* copy of *hb* driven by the more *D. melanogaster*-like CRM and the *D. melanogaster* copy of *hb* driven by the more *D. simulans*-like CRM (purple). G) Allele-specific expression measured by pyrosequencing in slices from 4 wild-type hybrid and 3 allele-swapped hybrid embryos. Error bars indicate standard deviation across 3 SNPs in all slices at the indicated slice. Significance markers indicate results of 2-sample, 2-sided *t*-tests. As usual, +1 is 100% *D. simulans* bias, and -1 is 100% *D. melanogaster* bias.

To test the prediction that the regulatory changes at this locus were responsible for the allele-specific expression, we used CRISPR-Cas9 and homology-directed repair genome editing to introduce the SNP cluster containing the Bicoid/Hkb binding site from *D. simulans* into *D. melanogaster* embryos [49, 50]. Sanger sequencing of the region showed no off-target edits. When we stained *hb* in homozygous embryos, the patterns were qualitatively quite similar (Fig 4D and 4E and S12 Fig), as expected based on our comparison of the *D. melanogaster* and *D. simulans* atlases (Fig 2B and 2C).

Since the *in situ* hybridizations could be influenced by even slight mismatches in the developmental timing of each embryo, to test the effects of the Bicoid/Hkb binding site SNP we generated a hybrid *D. melanogaster* × *D. simulans* embryo with the regulatory alleles swapped. Since both alleles in a hybrid are in the same nuclei, developmental timing of their expression is perfectly matched.

To generate this allele-swapped hybrid, we took advantage of natural variation within the *D. simulans* population. We noticed that of the two SNPs that differ between *D. melanogaster* and *D. simulans* *w*^501^ in site 1, the SNP that is outside of the core Bcd and Hkb binding motifs is fixed in a survey of 20 *D. simulans* lines, whereas the SNP within the core of the motif (position 4,520,429; Fig 4A) is segregating in *D. simulans* and is the minor allele (present in 3 of the 20 lines in [51]). We then screened a number of *D. simulans* stocks and found a naturally occurring line of *D. simulans* that had the *D. melanogaster*-like sequence in the core motif [52]. Sanger sequencing of the rest of the CRM did not reveal any unexpected SNPs. We crossed this naturally-occurring *D. melanogaster*-like *D. simulans* with the *D. simulans*-like *D. melanogaster* line that we generated with gene editing. We then sliced mid-stage 5 embryos at the same stage as in the RNA-seq experiments, and performed pyrosequencing to determine ASE in *hunchback* transcription. We used 3 allele-swapped hybrid embryos and 4 “wild-type” hybrid embryos. As expected, the *D. melanogaster* bias in slice at the anterior tip of the wild-type embryo was partially reversed in the allele-swapped embryos (Fig 4F). By the 3rd slice into the embryo (approximately 42 μm from the anterior tip) this bias decayed below significance, and by the fifth slice the mean ASE was identical. Thus, we conclude that swapping the alleles affects *hb* ASE, and only in the anterior tip of the embryo, consistent with our prediction.

## Discussion

The study of allele-specific expression in F1 hybrids is a powerful tool for probing the evolution of gene expression [53, 54]. However, previous studies of *Drosophila* hybrids have been limited in their ability to pinpoint the causal variants responsible for the observed cis-regulatory divergence [11, 55, 56]. In particular, the use of adult samples comprising multiple cell types meant that there was comparatively little information about the regulatory environment. In contrast, by focusing on the *Drosophila* embryo and using spatially-resolved samples, we were able to leverage decades of research on *Drosophila* development [31–33, 37, 57–59]. Combining this information with mathematical modeling of gene expression patterns yielded specific, testable predictions about which sequence changes produced the observed expression differences (Fig 3). Finally, by using CRISPR-mediated genome editing, we were able to directly confirm the genetic basis of the divergence in *hb* expression.

Only a spatially resolved approach is likely to find all genes with cis-regulatory differences. Changes in the position but not the absolute level of expression would be lost in bulk samples, and spatially restricted expression changes would tend to be washed out by more highly expressed and less variable regions. Even though the slices do not perfectly align with segmentation gene boundaries, the slicing approach is currently the only way to generate samples with both enough spatial resolution to assay a *Drosophila* embryo and enough read depth for assaying ASE. However, advances in the resolution of spatial transcriptomics or the sequencing depth of single-cell approaches show great promise for studying the evolution of spatially-varying gene expression [60, 61]. Alternatively, samples from later stages in development might use the expression of cell-type specific markers to select for cell types of interest, although this approach has not been used in the syncytial blastoderm [62, 63].

A previous study found allele-specific expression for ∼ 15% of genes in a *D. melanogaster* × *D. simulans* hybrid adult [11]. Considering that 400-600 genes have anterior-posterior (AP) expression patterns in blastoderm stage embryos [28, 57], our results suggest that a roughly similar fraction of these patterned genes have strong svASE. We chose to restrict our study to the AP axis because it is straightforward to generate well-aligned slices with the long axis of a prolate object; there are no doubt many genes with dorsal-ventral expression differences as well, especially since DV CRMs tend to be shorter, and thus potentially more sensitive to sequence perturbation than AP CRMs [64].

Although our experiment with editing the *hunchback* locus was unable to completely resolve the molecular mechanism underlying the ASE, it does suggest that Bicoid may lose its activator activity at the anterior tip of the embryo. Although [40] found that the two Bicoid terms have a net negative effect in the anterior tip of the embryo for *eve*, in our model the balance of the linear activation term and the quadratic repression term is such that at the anterior tip the two approximately cancel each other out. This is consistent with the observations that torso signaling phosphorylates Bicoid in the anterior and deactivates it [65, 66], rather than making Bicoid function as a transcriptional repressor. However, it is not obvious how increased binding of an inactive factor would reduce expression. This suggests that Huckebein binding may be responsible for the ASE in the anterior tip of the embryo, despite the marginal agreement with the consensus motif. Although the motif hit for Hkb was very weak—especially in *D. melanogaster*—Hkb was previously found to bind this locus in *D. melanogaster* embryos [37]. Furthermore, it is unclear what level of agreement with the consensus motif represents biologically relevant binding, especially since TF binding is modulated by chromatin accessibility. Sensitive chromatin immunoprecipitation studies might distinguish between Bicoid and Huckebein action in the anterior tip, but the small number of nuclei involved would make these experiments especially challenging.

The *hb* CRM causal variant’s lack of any obvious phenotypic effect is not surprising, since development at this stage has been shown to be highly robust to perturbations. For instance, although embryos across a 6-fold range in Bicoid concentrations have widespread downstream transcriptional changes, development is able to buffer these changes, at least in part due to differential apoptosis at later stages [67–70]. Development is also robust to large variation in the amount of *hunchback*, with hemizygous embryos giving rise to phenotypically normal adults [71], and previous studies have also found subtle variation in *hunchback* expression patterns between *D. melanogaster* and either *D. virilis* or *D. yakuba*, but these changes have also not been linked to phenotypic divergence [72, 73]. It is also possible that the reduced *hb* expression in *D. simulans* matters only in particular stress conditions, but given the similar cosmopolitan geographic distributions of *D. melanogaster* and *D. simulans*, it is not obvious what conditions those might be. Looking ahead, it will be interesting to apply svASE to other species and developmental timepoints, to efficiently pinpoint candidate genes underlying phenotypic divergence. For example, applying a conceptually similar approach of tissue-specific ASE to the oenocytes of adult *D. simulans* × *D. sechellia* hybrids revealed a gene required for inhibition of interspecies mating [74].

We believe that the informed modeling approach we have taken can serve as a template for dissecting other cis-regulatory modules. For example, eight genes with clear svASE are present in the BDTNP expression atlas [31], and preliminary modeling of the four genes without pair-rule-like striping patterns suggested plausible binding site changes that could be responsible (S13 Fig). In some of these cases, there are multiple binding site changes that could explain our observed svASE equally well, but predict different dorso-ventral gene expression patterns in *D. simulans*—in these cases, *in situ* hybridization for the gene with svASE should provide clearer hypotheses of the causal variants. While more complex approaches might be applied to model the enhancer more faithfully [9, 75, 76], the ability of the simple logistic model to make useful predictions is remarkable. This approach, when applied more broadly and in concert with evolutionary studies, may help refine our understanding of the molecular mechanisms of the cis-regulatory logic underlying spatial patterning.

## Materials and methods

### Strains and hybrid generation

Unless otherwise indicated, we used DGRP-340 as the *D. melanogaster* strain, and *w*^501^ as the *D. simulans* strain. Males of both species were co-housed for 5 days at 18C in order to improve mating efficiency, then approximately twenty males were mated with ten 0–1 day old virgin females of the opposite species per vial with the stopper pressed almost to the bottom. After cohousing, males were sorted using eye color as a primary marker. 5 days later, flies from the vials with larvae were put into a miniature embryo collection cage with grape juice-agar plates and yeast paste (Genessee Scientific).

### RNA extraction, library preparation, and sequencing

We selected single embryos at the target stage (based on depth of membrane invagination) on a Zeiss Axioskop with a QImaging Retiga 6000 camera and transferred them to ethanol-cleaned Peel-a-way cryoslicing molds (Thermo Fisher). We then applied approximately 0.5 μL of methanol saturated with bromophenol blue (Fisher Biotech, Fair Lawn N.J.), then washed with clean methanol to remove the excess dye. Next, we covered the embryo in Tissue-Plus O.C.T Compound (Fisher Healthcare) and froze the embryo at -80 until slicing. We sliced the embryos using a Microm HM550 cryostat, with a fresh blade for each embryo to minimize contamination. We used 1mL of TRIzol (Ambion) with 400 μg/mL of Glycogen (VWR) to extract RNA, ensuring that the flake of freezing medium was completely dissolved in the TRIzol.

Next, we randomized the order of the RNA samples (see Fig 1—source data 1), then prepared libraries using a slightly modified version of the SMART-seq2 protocol [18]. As described in [19], instead of steps 2-5 of the protocol in [18], we added 1μL of oligo-dT and 3.7μL of dNTP mix per 10μL of purified RNA; in step 14, we reduced the pre-amplification to 10 cycles; from step 28 onwards, we reduced the volume of all reagents by five-fold; and at step 33, we used 11 PCR amplification cycles.

We sequenced libraries in 4 separate lanes on either an Illumina HiSeq 4000 or an Illumina NextSeq (See S1 File for lane and index details). RNA-seq data is available from the Gene Expression Omnibus with accession GSE102233.

For pyrosequencing, we generated cDNA from the RNA using SuperScript II and random hexamers. We then amplified a 167 bp amplicon across 4 SNPs using primers AGCTGGACGCCGTCGAAC and 5’ biotinylated GCAACTGAAAGTACCCAGCACAC with DreamTaq Green Master Mix (Thermo Fisher). Then, we performed pyrosequencing using sequencing primer CACATGGGCCGTCTC on a Qiagen Q24 Pyrosequencer according to manufacturer instructions.

### Sequencing data processing and ASE calling

In order to call mappable SNPs between the species, we used Bowtie 2 (version 2.2.5, arguments --very-sensitive) [77] to map previously published genomic sequencing data for the lines in this study (SRR835939, SRR520334 from [78, 79]) onto the FlyBase R5.57 genome. We then used GATK (version 3.4-46, arguments -T HaplotypeCaller-genotyping_mode DISCOVERY--output_mode EMIT_ALL_SITES-stand_emit_conf 10-stand_call_conf 30) to call SNPs [80].

Next, we created a version of the *D. melanogaster* genome with all SNPs that are different between the two species masked. We used STAR (version 2.4.2a, arguments --clip5pNbases 6) to map each sliced RNA-seq sample to the masked genome [81]. We further filtered our list of SNPs to those for which, across all the RNA-seq samples, there were at least 10 reads that supported each allele. We then implemented the WASP filtering step for reads that did not remap to the same location upon computationally reassigning each SNP in a read to the other parent as described in [82]. Although we mapped only to a *D. melanogaster*-based genome, in our experience the choice of reference genome has relatively small effects when looking for patterns of ASE between samples, especially after using the WASP pipeline to filter out reads that can only map reliably in one species [74].

To call ASE for each sample, we used the GetGeneASEbyReads script in the ASEr package (available at https://github.com/TheFraserLab/ASEr/, commit cfe619c69). Briefly, each read is assigned to the genome whose SNP alleles it matches. Reads are discarded as ambiguous if there are no SNPs, if there are alleles from both parents, or if the allele at a SNP does not match either parent. We excluded samples with fewer than 1 million reads (6 samples) or an overall mapping rate of <52% (9 samples), as we found samples below these cutoffs had much noisier ASE data. Additionally, ASE is ignored if the gene is on the X chromosome and the slice came from a male embryo (which only have an X chromosome from their mother). All other analysis scripts are available at https://github.com/TheFraserLab/HybridSliceSeq (commit b3b8e06, doi:10.5281/zenodo.1193784).

To assign bias in overall ASE, we used DESeq2 to determine whether there was a difference in the number of *D. melanogaster* reads versus the number of *D. simulans* reads. We corrected the size factors for each column containing reference or alternate reads to be equal to the sum of the size factors for the column with reference allele counts and the column with alternate allele counts. Then, we performed DESeq using default arguments and design matrix ∼Sample + refalt. To account for the different number of samples with each mother and the possibility of differing levels of maternal deposition, we performed DESeq separately on samples from each direction of the cross (i.e. *D. melanogaster* mother and *D. simulans* mother). We then plotted the DESeq-estimated log_2_ fold changes for genes that [23] called as either maternal, maternal-zygotic, or zygotic in S5 Fig. To call a gene as either *D. melanogaster* or *D. simulans* biased (S7 Fig), we required a log_2_ fold change indicating bias towards the respective parent and FDR< 0.05 in both directions of the cross.

### Earth mover distance and spatial patterning differences

Earth mover distance (EMD), as described in [20], is a non-parametric metric that compares two distributions of data in a way that roughly captures intuitive notions of similarity. It represents the minimal amount of work (defined as the amount moved multiplied by the distance carried) that must be done to make one pattern equivalent to another, as if transporting dirt from one pile to another. For each slice, we calculate the absolute expression of each gene using cufflinks v.2.2.1 [83]. We normalize all absolute expression patterns by first adding a constant amount to mitigate noise in lowly expressed genes, and then by dividing by the total amount of expression in an embryo.

To compare between the hybrids and the parental embryos, we first calculated a spline fit for each gene on each of the parental embryos separately, first smoothing by taking a rolling average of 3 slices. We then fit a univariate spline onto the smoothed data using the Scipy “interpolate” package. Then, we recalculated the predicted expression for a hypothetical 27-slice embryo of each parent, then averaged the expression data. We next calculated the EMD between this simulated averaged embryo and each of the hybrid embryos. For each gene, we then performed a one-sided t-test to determine whether the hybrid embryos were more different from the average than the EMD between the parental embryos. Although 342 genes had a nominal p-value < .05, none of these remained significant after Benjamini-Hochberg multiple hypothesis testing correction [84]).

To compare embryos between directions of the cross, we calculated the pairwise EMD between embryos within a direction of a cross (i.e. the three possible pairs of hybrid embryos with a *D. melanogaster* mother and the pair of embryos with the *D. simulans* mother) and the pairwise EMD between hybrid embryos with different parents (e.g. the first replicate of embryos). We then used a one-sided t-test to determine whether the EMDs were larger between groups than within. Benjamini-Hochberg FDR estimation yielded 171 genes with a q-value less than.05, whereas Bonferroni p-value correction yielded 12 genes at *α* < .05 [84]).

### Identification of allele-specific expression patterns

To call svASE, we fit a 4-variable least-squares regression of either a sigmoidal logistic function (*f*(*x*) = *A*/(1 + exp(*w*(*x* − *x*_0_))) − *y*_0_) or a peak-like Gaussian function (*f*(*x*) = *A* ⋅ exp(−(*x* − *x*_0_)^2^/*w*^2^) − *y*_0_). We then considered any gene where the fit explained at least 45% of the variance ($R^{2}=\sum {\left(A_{i}-f\left(x_{i}\right)\right)}^{2}/\sum {\left(A_{i}-\bar{A}\right)}^{2}$, where *A*_*i*_ is the ASE value in the *i*th slice, and $\bar{A}$ is the average ASE value for that gene) as having svASE.

To calculate a false discovery rate, we shuffled the columns (i.e. the spatial coordinates) of the ASE matrix 1,000 times. For each of the shuffles, we fit both of the ASE functions. Most of the shuffled matrices yielded no fits that explained at least 45% of the variance, only a handful of the matrices yielded a single gene that cleared the threshold, and no shuffled matrix had two or more genes that cleared the threshold.

### *In situ* atlas comparison and bias calculation

Because the different expression atlases had different background levels for each gene and in each species, we normalized expression for *hunchback* by subtracting the mean expression in the inter-stripe region between 55% and 75% embryo length, then normalizing by dividing the expression in the *D. melanogaster* and *D. simulans* atlas by the expression value at the 90th percentile of nuclei not in the inter-stripe region. For each nucleus in the *D. melanogaster* atlas, we found the most similar nucleus in the *D. simulans* atlas by using an approach very similar to that in [12]: of the 30 physically closest nuclei, we selected as the “best” nucleus the one that minimized the sum of the squares of the differences in expression for each gene in both atlases. For each *D. melanogaster* nucleus, we then computed the expected bias using Eq 1 (Fig 2D). For Fig 2E, we grouped the nuclei by x-coordinate to simulate slicing, then combined the expression of each nucleus *i* in each slice *s* in an analgous manner to Eq 1:

$$\begin{array}{c} ASE_{predicted}=(\sum_{i\in s}f_{sim}\left(i\right)-\sum_{i\in s}f_{mel}\left(i\right))/(\sum_{i\in s}f_{sim}\left(i\right)+\sum_{i\in s}f_{mel}\left(i\right)) \end{array}$$ (2)

We then computed the Pearson correlation of the predicted and real ASE values.

### Identification of binding site changes and predicted effects on hybrid embryos

For *hunchback* we used the coordinates for the regulatory elements as defined in the RedFly database to extract the sequence of each regulatory region from the reference sequence files [32]. For the other genes whose regulatory programs we investigated for causal binding changes, we used Bedtools to find any non-exonic DNase accessible region within 15,000 bp of each gene [85, 86]. We then used BLAST v2.3.0+ to search for the orthologous region in *D. simulans*. We combined motifs from the databases in [59, 87–90] by taking the most strongly-supported motif for a given TF, then we used the FIMO tool of the MEME suite to search for binding sites for all TFs with known spatial patterns [38, 91]. We also included the motif for hkb from the Fly Factor Survey, which had the hits shown in Fig 3 [92].

In order to construct a model of transcription regulation for the other genes with svASE and simple expression patterns in the [31] atlas, we built models that contained the TFs with binding changes for the target gene as well as up to 4 other TFs with localization data in the [31] atlas and known roles as patterning factors during early development (i.e. Bcd, Gt, Kr, *cad, tll, D, da, dl, kni, mad, med, shn, sna, twi, zen, brk, emc, numb, rho, tkv* and *Doc2*); when available, we used protein localizations instead of RNA *in situ* hybridization (i.e. for Bcd, Gt, and Kr). For a given combination of factors, we used the Python Statsmodels package to fit a logistic regression to the anterior stripe of *hunchback* [93]. In line with the procedure in [40], we separated the two *hunchback* expression domains and fit the data on nuclei with either the anterior stripe or no *hunchback* expression. We then selected the best model based on fraction of variance in the original data explained by the fit.

To estimate the likely effect of each transcription factor change, we adjusted the relevant parameter(s) in the model by a range of values (see S11 Fig). We then generated predicted svASE by predicting expression in each nucleus under the original model and the model with the relevant parameter(s) changed, then calculated predicted svASE using Eq 2. In general, both using Pearson correlation and measuring the fraction of the variance in the real ASE explained by the predicted ASE suggested the same direction of change to the coefficient, although the absolute magnitude of change that yielded the “best” result may have been different.

### Genome editing and screening

We inserted the *D. simulans* SNPs into *D. melanogaster* using CRISPR-Cas9 directed cutting followed by homology directed repair [49]. We inserted the gRNA sequence GGT ACA GGT CGC GGA TCG GT into pU6-bbsI (a generous gift from Tim Mosca and Liqun Luo). We injected the plasmid and a 133bp ssDNA HDR template (IDT, San Diego, CA) into y[1] Mvas-Cas9ZH2A w[1118] embryos (Bloomington Stock #51323, BestGene Inc, Chino Hills, CA). The edited sequence affects a recognition sequence for the restriction enzymes BsiE1 and MspI (New England Biolabs) which specifically cut the *D. melanogaster* and *D. simulans* sequences, respectively. We screened putatively edited offspring by PCR amplifying a region around the *hunchback* anterior CRM (primers CGT CAA GGG ATT AGA TGG GC and CCC CAT AGA AAA CCG GTG GA) then cutting with each enzyme separately. Presumptively edited lines were then further screened via Sanger sequencing.

For the *in situ* hybridization, we generated DIG-labeled antisense RNA probes by first performing RT-PCR on *D. melanogaster hunchback* cDNA using primers with a T7 RNA polymerase handle (AAC ATC CAA AGG ACG AAA CG and TAA TAC GAC TCA CTA TAG GGA GA), then creating full-length probes with 2:1 DIG-labeled UTP to unlabeled UTP [94]. We then performed *in situ* hybridization in 2-4 hour old embryos of each strain according to a minimally modified, low-throughput version of the protocol in [94] (dx.doi.org/10.17504/protocols.io.g7bbzin). Stained embryos were imaged on the Zeiss Axioskop above.

## Supporting information

S1 Table Summary data for embryos used. (XLSX)

S2 Table Coefficients of the best-fit model for TFs bound near the anterior activator of *hb*. (XLSX)

S3 Table Summary data for embryos used for pyrosequencing. (XLSX)

S1 Fig Correlation of expression between adjacent slices. Log-log plots of expression between each slice and the closest slice from any embryo. Pearson correlation is indicated in the box.
(TIF)

S2 Fig Using earth mover distance to identify genes with different expression patterns between the hybrids and the parents. A) We used earth mover distance (EMD) to quantify the difference in patterns between each embryo. Given the green and pink patterns, EMD minimizes the amount of work that must be done to turn one pattern into the other. B) Hypothetical examples of pattern differences with low, intermediate, and high EMDs. C) Histograms of replicate hybrid embryos compared to each other (dark blue) and hybrid embryos compared to the average of splines fit on the parental embryos (cyan).
(TIF)

S3 Fig Using earth mover distance to identify genes with different expression patterns between the directions of the hybrid cross. We found 171 genes with a significantly different EMD between each direction of the cross compared to replicates of each direction (Benjamini-Hochberg q-value <.05; [84]). The heatmap for each gene has each embryo aligned with anterior to the left and posterior to the right. Genes that are also significant after Bonferroni multiple testing correction are marked in red. We manually categorized these as due either to A) the embryos having clear parent of origin expression patterns that we interpret as due to species-specific maternal deposition (ASE data for these genes generally support this interpretation), B) a single embryo having a different expression pattern, marked with a red star, or C) more subtle expression differences or noise in expression measurement. Order within each class is arbitrary. Of the 52 genes with differences in maternal deposition, 39 were annotated with the GO term “binding” (GO0005488, GOTerm Finder corrected p-value 2.6 × 10^−7^, [95]), though functional importance of these changes, if any, are unclear.
(TIF)

S4 Fig Counts of maternal and paternal reads for each sample. Each point represents read counts from a single sample. There are approximately 6.8 fold more reads mapping to the maternal genome (x-axis) than the paternal (y-axis) due to the significant complement of maternally deposited reads. There is no obvious contribution of the direction of the cross (i.e. samples with a *D. melanogaster* mother vs a *D. simulans* mother) to the rate of calling paternal reads, suggesting that the WASP pipeline has adequately controlled for mapping bias. Assuming that the paternally mapping reads account for approximately half of the zygotically expressed transcripts, there are approximately 2.9 fold more maternally deposited transcripts than zygotically expressed ones.
(TIF)

S5 Fig Violin plots of log_2_ fold change of the maternal allele compared to the paternal allele for genes in the three categories determined by [23]. We used DESeq to estimate average log_2_ fold changes between the maternal and paternal alleles. We filtered out genes with fewer than 20 ASE counts in at least half of the samples, then made violin plots showing the distribution of the average log_2_ fold change for Maternal (mat), Maternal-zygotic (matzyg), and zygotic (zyg) genes, as called by [23]. Numbers of genes with measurable ASE in at least half of the samples are indicated below each category. Black hashes indicate values for each individual gene, and the blue bar indicates the median log_2_ fold change.
(TIF)

S6 Fig Complete heatmap of ASE for genes with svASE. Genes from Fig 1A and 1C in the same order, but with the complete set of ASE data and *R*^2^ values of the fit provided. A) Genes best fit by a logistic function and B) genes best fit by a normal function.
(TIF)

S7 Fig Genes with species-specific expression, regardless of parent of origin. Genes strongly biased towards transcribing *D. melanogaster* (A) or *D. simulans* (B) alleles, regardless of whether *D. melanogaster* or *D. simulans* is the mother or father. Absolute expression values are normalized to the most highly expressed slice in each embryo (or 10 FPKM, whichever is higher). Genes are sorted by highest FPKM in the species that is un-expressed in the hybrid. The column (sim-mel)/(sim+mel) is the expected ASE assuming expression level is encoded in cis, and is computed by comparing matching slices of the parental embryos. ASE is not interpolated if there are not enough reads to call in a given slice.
(TIF)

S8 Fig The *D. melanogaster* biased expression in the anterior tip persists across all time points except the last in the atlases. Absolute expression and computed bias per nucleus and per slice at various stages throughout embryonic development. Correlation indicates the Pearson correlation of computed bias with the true ASE, binned by an equal fraction of the embryo as each slice. All stages except the late 76-100% invagination show a *D. melanogaster* bias in the anterior tip. As expected, the time points closest to the stage we measured (approximately 50-65% membrane invagination) have the highest correlation, while the earliest and latest time points have lower correlation with the observed ASE.
(TIF)

S9 Fig Motif content of the CRMs for all TFs included in the model. Positions of TF binding motifs in the canonical anterior CRM from [33] (A), the distal “shadow” CRM from [35] (B), and the non-minimal 2.4kb CRM construct from (of which the canonical CRM is a subset) [34], split across two lines for compactness. Within each CRM, the top line indicates the location of SNPs (colored lines) and insertions/deletions (grey bars on the side with the insertion) in a pairwise alignment of the two sequences. The middle track indicates DNase accessibility from [86]. The third track indicates the locations of FIMO motifs for a variety of TFs. TFs that have a motif with approximately equal strength (±20%) within 5bp have reduced opacity to better highlight motif changes. Bar height corresponds to FIMO score.
(TIF)

S10 Fig Prediction of the best-fit model for *hb* expression. The posterior stripe of *hb* expression was removed prior to the fitting process. McFadden’s Pseudo *R*^2^ as reported by the statsmodels Python package.
(TIF)

S11 Fig Correlation of the predicted *hb* ASE with the real ASE (A) and percent of the variance explained by predicted ASE (B) at a range of coefficient strengths. We altered each coefficient separately (with the exception of the Bicoid terms, which we also adjusted in tandem) by multiplying by a range of multipliers, then predicting ASE. Although increasing the *kni* term in the model had the best correlation with the real ASE, there were no Kni motif changes in the known CRMs, so we excluded it from consideration. In addition, due to the buffering effects of the other TFs in the full model, we could not find a change that, when applied to both the Bcd and Bcd^2^ term that explained the ASE; however, adjusting a simpler model consisting of only terms for Bcd, Bcd^2^, *D*, and *twi* did yield a good fit. The actual predicted ASE for these models at a given change of coefficient is qualitatively very similar (C-D).
(TIF)

S12 Fig Staging of the embryos in Fig 4D and 4E based on depth of membrane invagination. In order to find closely staged embryos, we compared the depth of the cellular membrane invagination (A) in the inter-stripe region (marked in black boxes in B and C).
(TIF)

S13 Fig Proposed TF binding changes that generate svASE in *Ance*, *bmm*, *CG8147*, and *path*. Modeling suggests plausible changes to the regulatory function that could generate the observed allele-specific expression. We fit a logistic model to the atlas expression, then adjusted each term of the model to find the coefficient that best matches the observed ASE in the slices (after setting mean ASE to match in the real and predicted data, since there may be mapping bias). The expression is then predicted in the adjusted model (purple embryo), which is also used to generate predicted ASE on a per-nucleus (red/blue embryo) and computationally sliced (heatmap) basis. Multiple TF changes can generate substantially similar sliced ASE data, while still having distinct expression patterns;*in situs* of the *D. simulans* embryos would be needed to distinguish between them. We did not attempt modeling of the pair-rule genes *pxb*, *Bsg25A*, *comm2*, and *pxb*, since other pair-rule genes have multiple, independent regulatory elements, likely complicating the modeling approach.
(TIF)

S1 File Table of sequencing indices and batches. (TSV)

S2 File Table of ASE values in each slice. (TSV)

S3 File Table of absolute expression values in each slice, used for comparing patterning differences in S3 Fig. (TSV)
