# Supplementary figures and images for: Spatially varying cis-regulatory divergence in Drosophila embryos elucidates cis-regulatory logic
Source: PLoS Genet. 2018 Nov 1;14(11):e1007631. doi: 10.1371/journal.pgen.1007631 (PMC6211617; doi:10.1371/journal.pgen.1007631)

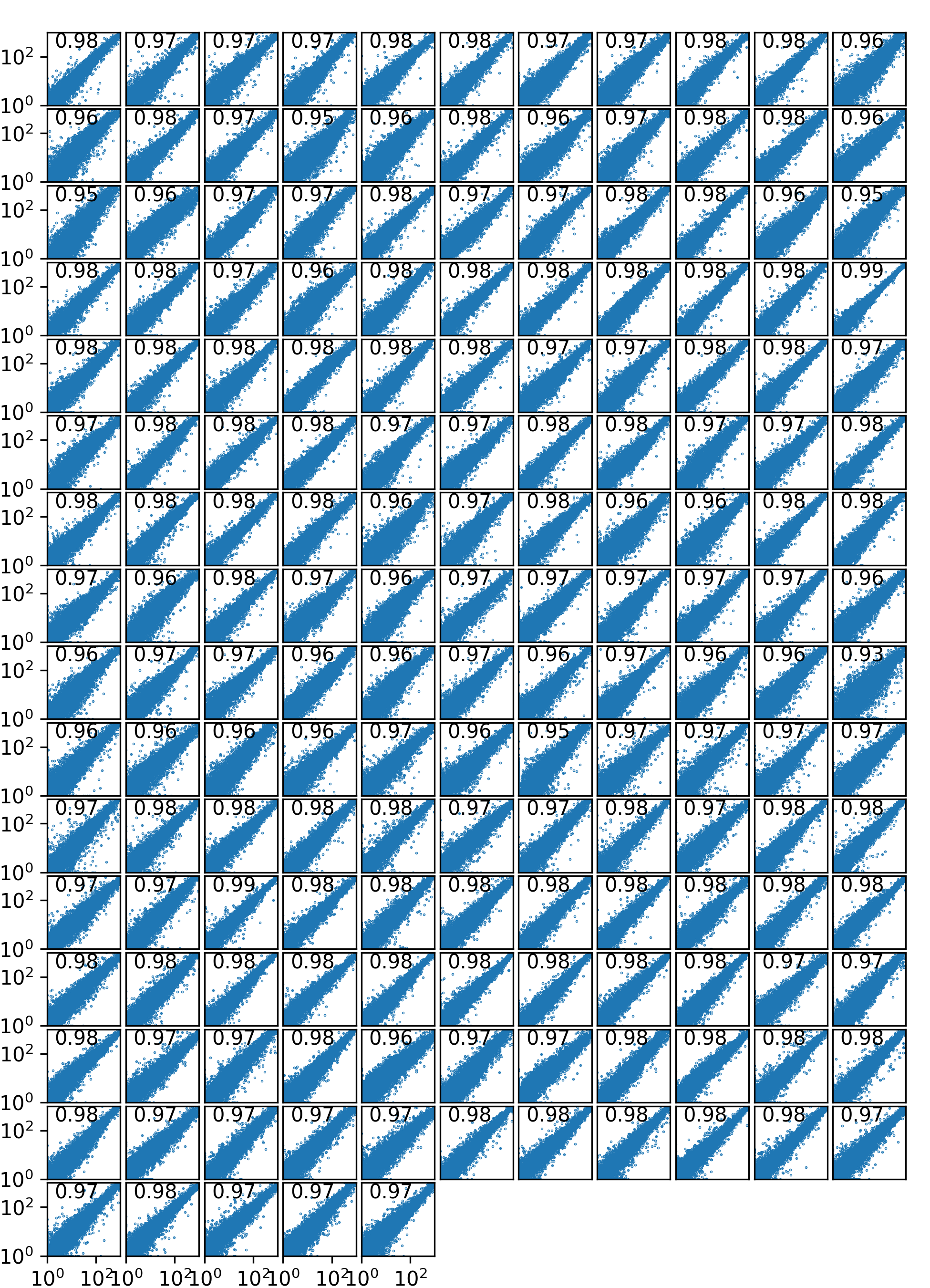

Supplement: S1 Fig — Log-log plots of expression between each slice and the closest slice from any embryo. Pearson correlation is indicated in the box. (TIF) [file pgen.1007631.s004.tif]

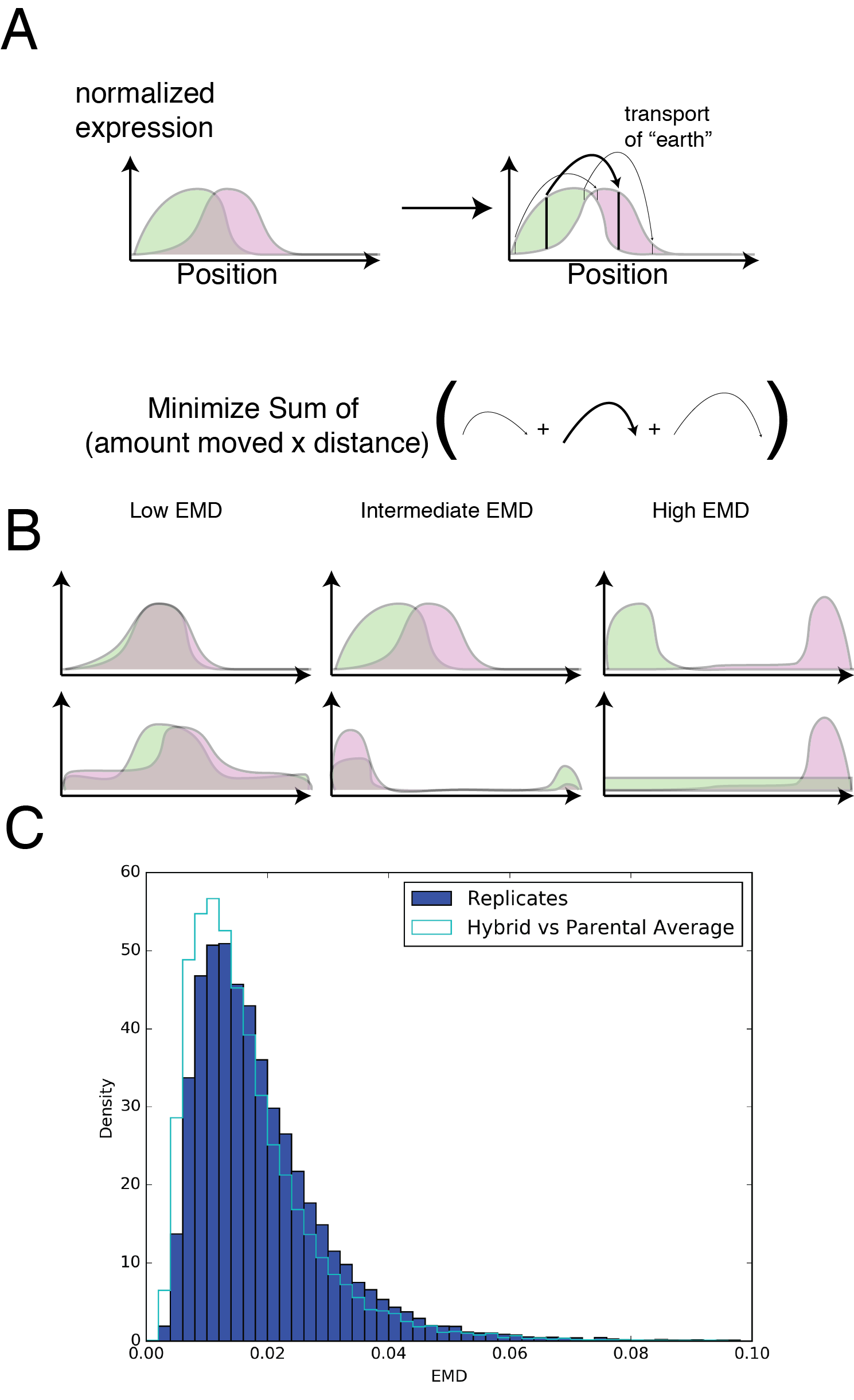

Supplement: S2 Fig — A) We used earth mover distance (EMD) to quantify the difference in patterns between each embryo. Given the green and pink patterns, EMD minimizes the amount of work that must be done to turn one pattern into the other. B) Hypothetical examples of pattern differences with low, intermediate, and high EMDs. C) Histograms of replicate hybrid embryos compared to each other (dark blue) and hybrid embryos compared to the average of splines fit on the parental embryos (cyan). (TIF) [file pgen.1007631.s005.tif]

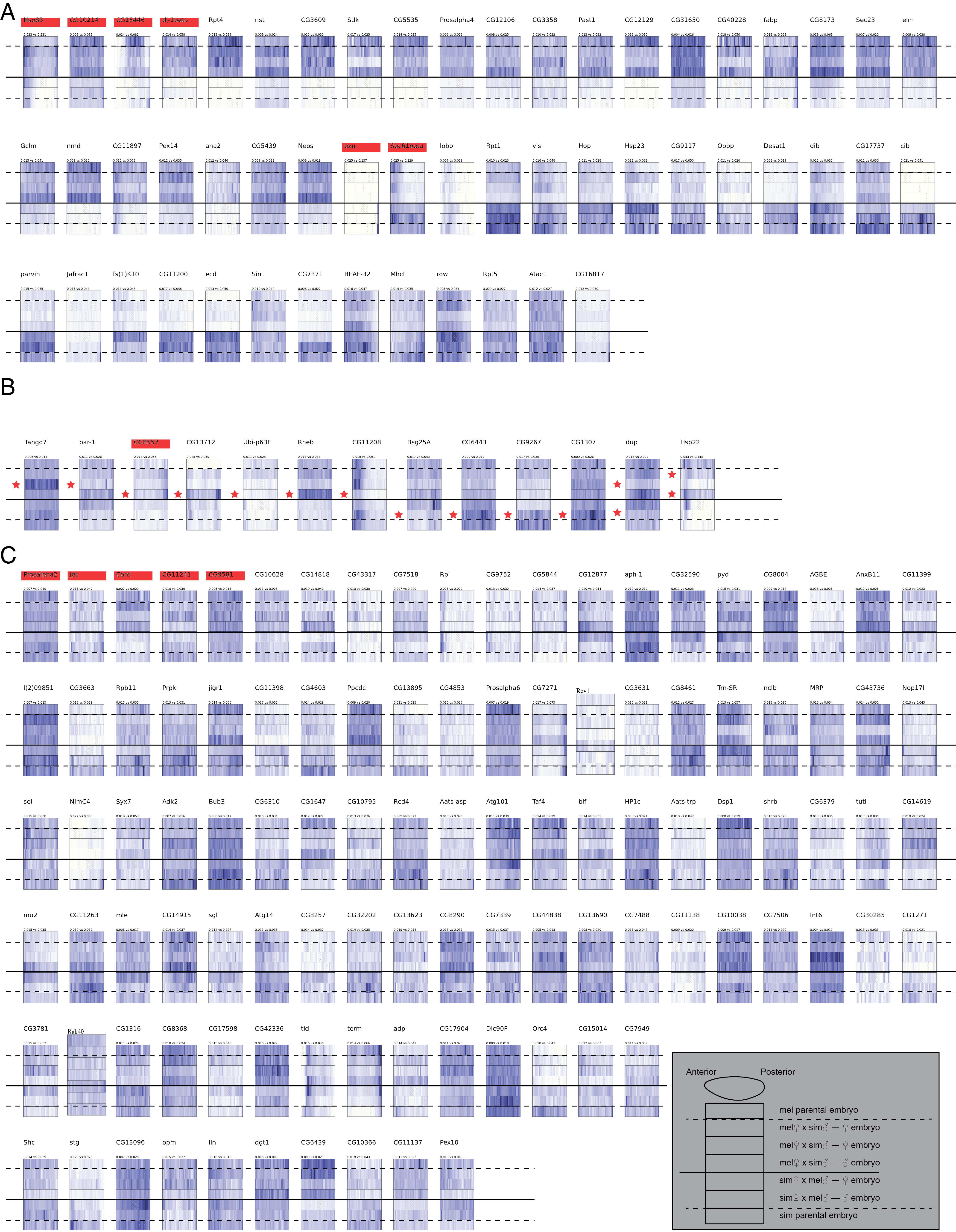

Supplement: S3 Fig — We found 171 genes with a significantly different EMD between each direction of the cross compared to replicates of each direction (Benjamini-Hochberg q-value <.05; [84]). The heatmap for each gene has each embryo aligned with anterior to the left and posterior to the right. Genes that are also significant after Bonferroni multiple testing correction are marked in red. We manually categorized these as due either to A) the embryos having clear parent of origin expression patterns that we interpret as due to species-specific maternal deposition (ASE data for these genes generally support this interpretation), B) a single embryo having a different expression pattern, marked with a red star, or C) more subtle expression differences or noise in expression measurement. Order within each class is arbitrary. Of the 52 genes with differences in maternal deposition, 39 were annotated with the GO term “binding” (GO0005488, GOTerm Finder corrected p-value 2.6 × 10^−7^, [95]), though functional importance of these changes, if any, are unclear. (TIF) [file pgen.1007631.s006.tif]

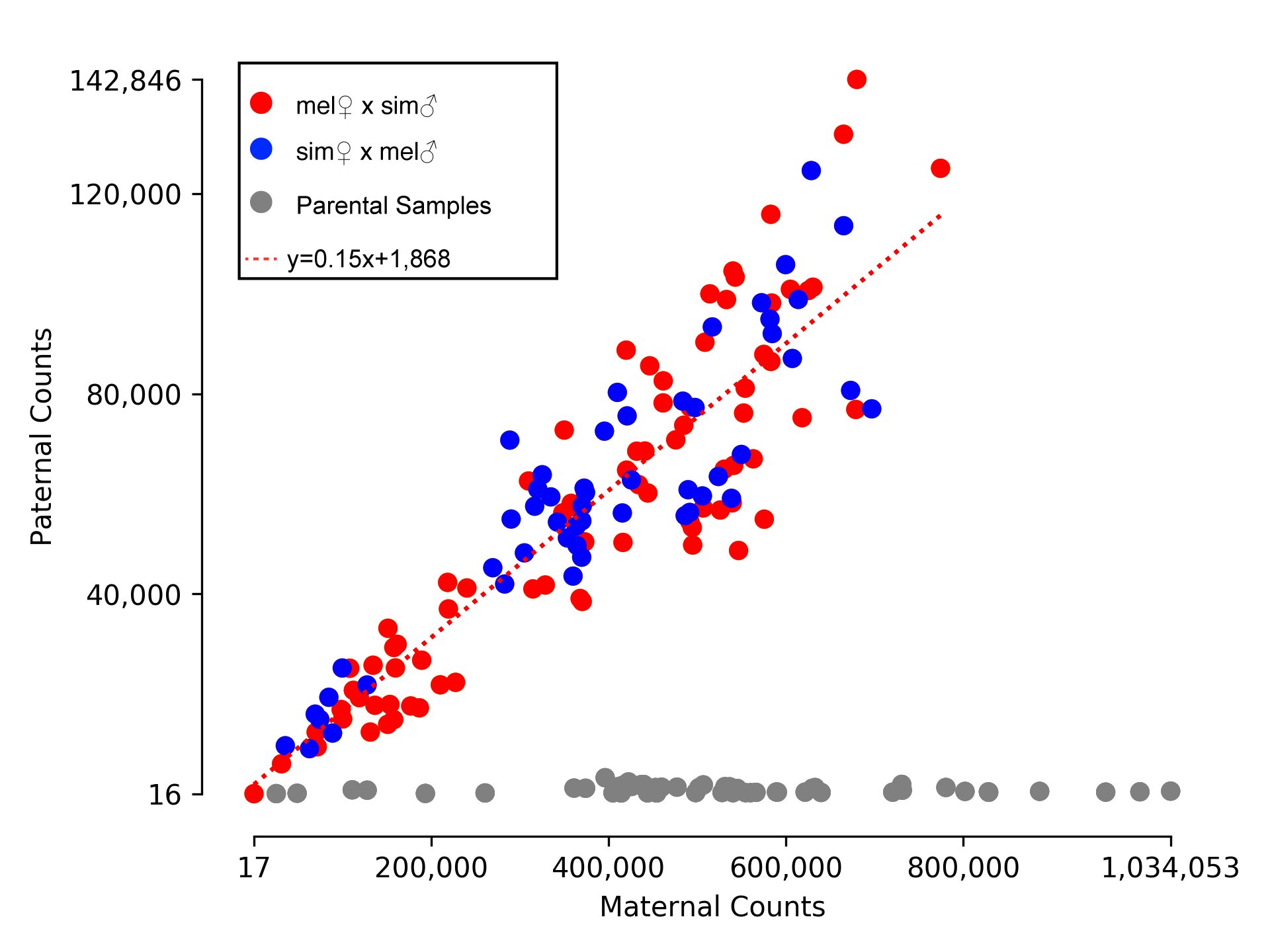

Supplement: S4 Fig — Each point represents read counts from a single sample. There are approximately 6.8 fold more reads mapping to the maternal genome (x-axis) than the paternal (y-axis) due to the significant complement of maternally deposited reads. There is no obvious contribution of the direction of the cross (i.e. samples with a D. melanogaster mother vs a D. simulans mother) to the rate of calling paternal reads, suggesting that the WASP pipeline has adequately controlled for mapping bias. Assuming that the paternally mapping reads account for approximately half of the zygotically expressed transcripts, there are approximately 2.9 fold more maternally deposited transcripts than zygotically expressed ones. (TIF) [file pgen.1007631.s007.tif]

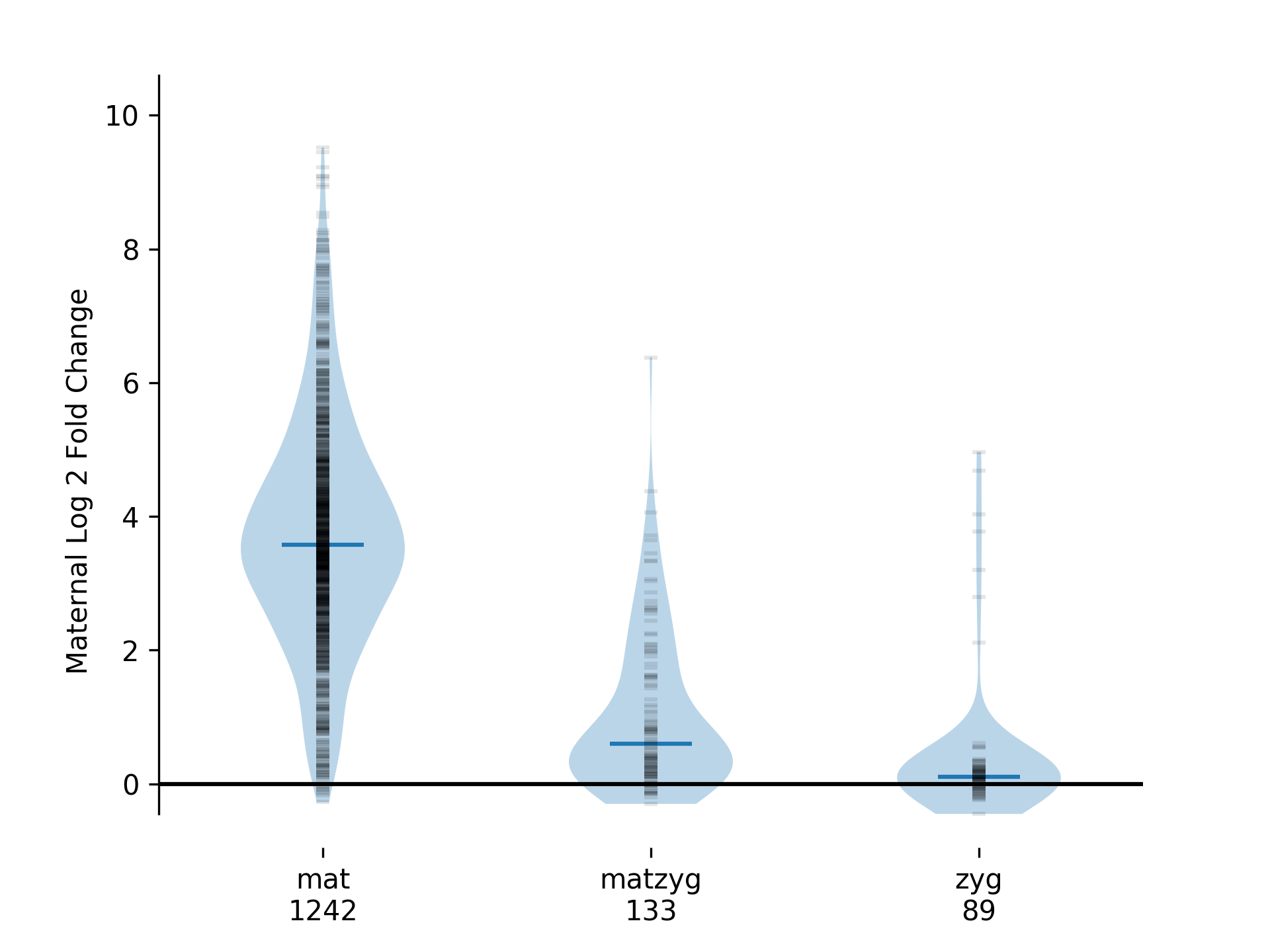

Supplement: S5 Fig — We used DESeq to estimate average log2 fold changes between the maternal and paternal alleles. We filtered out genes with fewer than 20 ASE counts in at least half of the samples, then made violin plots showing the distribution of the average log2 fold change for Maternal (mat), Maternal-zygotic (matzyg), and zygotic (zyg) genes, as called by [23]. Numbers of genes with measurable ASE in at least half of the samples are indicated below each category. Black hashes indicate values for each individual gene, and the blue bar indicates the median log2 fold change. (TIF) [file pgen.1007631.s008.tif]

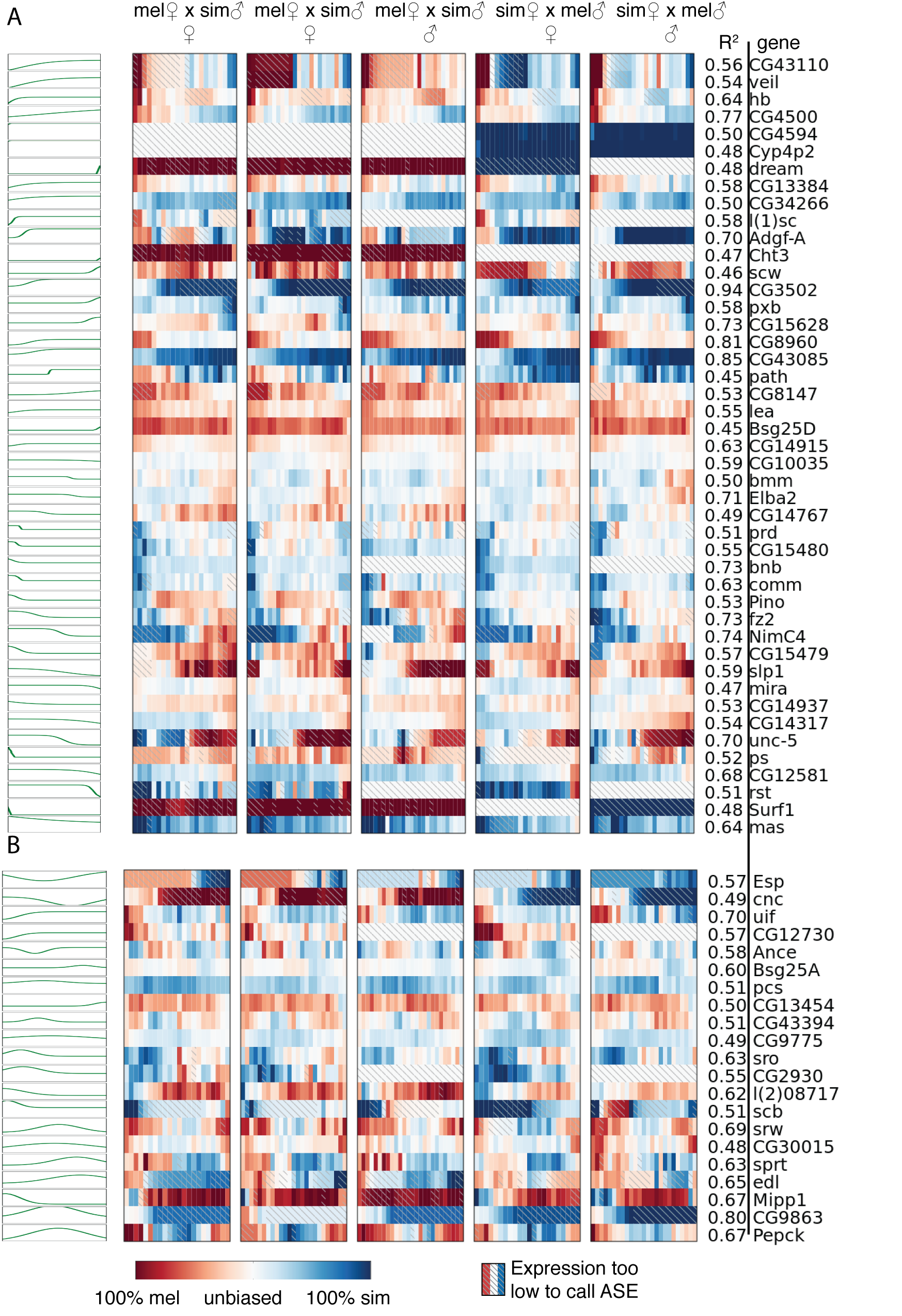

Supplement: S6 Fig — Genes from Fig 1A and 1C in the same order, but with the complete set of ASE data and R2 values of the fit provided. A) Genes best fit by a logistic function and B) genes best fit by a normal function. (TIF) [file pgen.1007631.s009.tif]

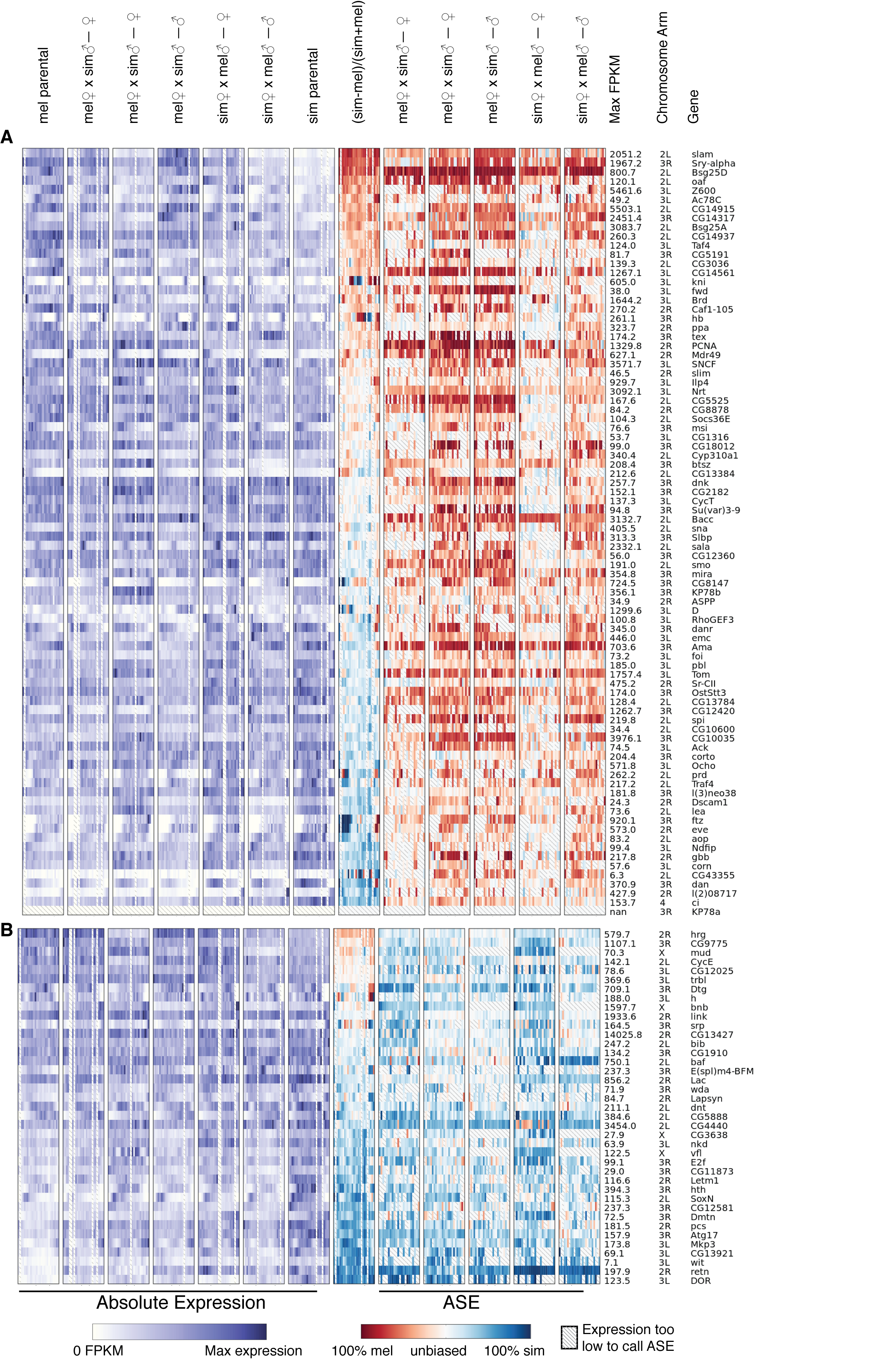

Supplement: S7 Fig — Genes strongly biased towards transcribing D. melanogaster (A) or D. simulans (B) alleles, regardless of whether D. melanogaster or D. simulans is the mother or father. Absolute expression values are normalized to the most highly expressed slice in each embryo (or 10 FPKM, whichever is higher). Genes are sorted by highest FPKM in the species that is un-expressed in the hybrid. The column (sim-mel)/(sim+mel) is the expected ASE assuming expression level is encoded in cis, and is computed by comparing matching slices of the parental embryos. ASE is not interpolated if there are not enough reads to call in a given slice. (TIF) [file pgen.1007631.s010.tif]

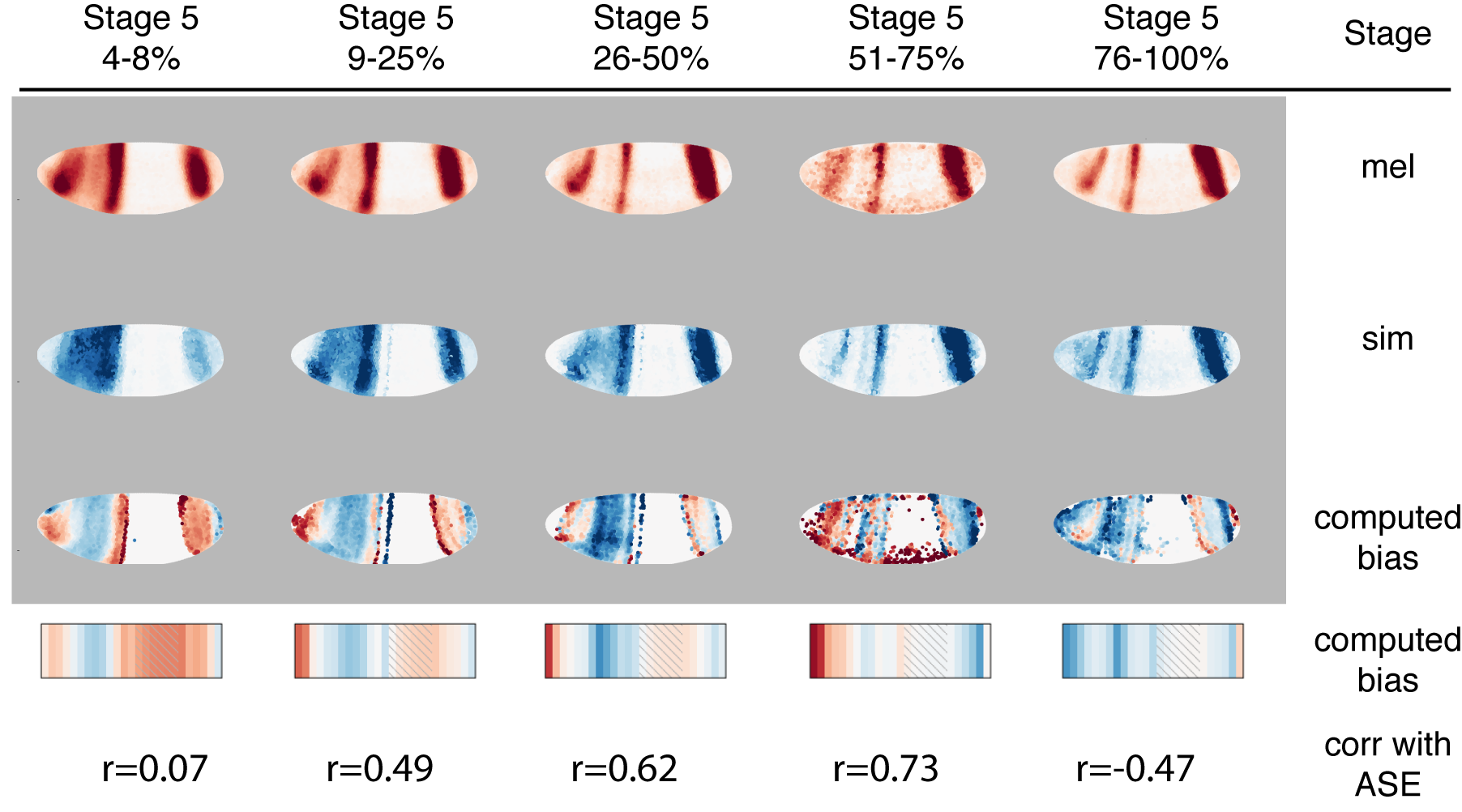

Supplement: S8 Fig — Absolute expression and computed bias per nucleus and per slice at various stages throughout embryonic development. Correlation indicates the Pearson correlation of computed bias with the true ASE, binned by an equal fraction of the embryo as each slice. All stages except the late 76-100% invagination show a D. melanogaster bias in the anterior tip. As expected, the time points closest to the stage we measured (approximately 50-65% membrane invagination) have the highest correlation, while the earliest and latest time points have lower correlation with the observed ASE. (TIF) [file pgen.1007631.s011.tif]

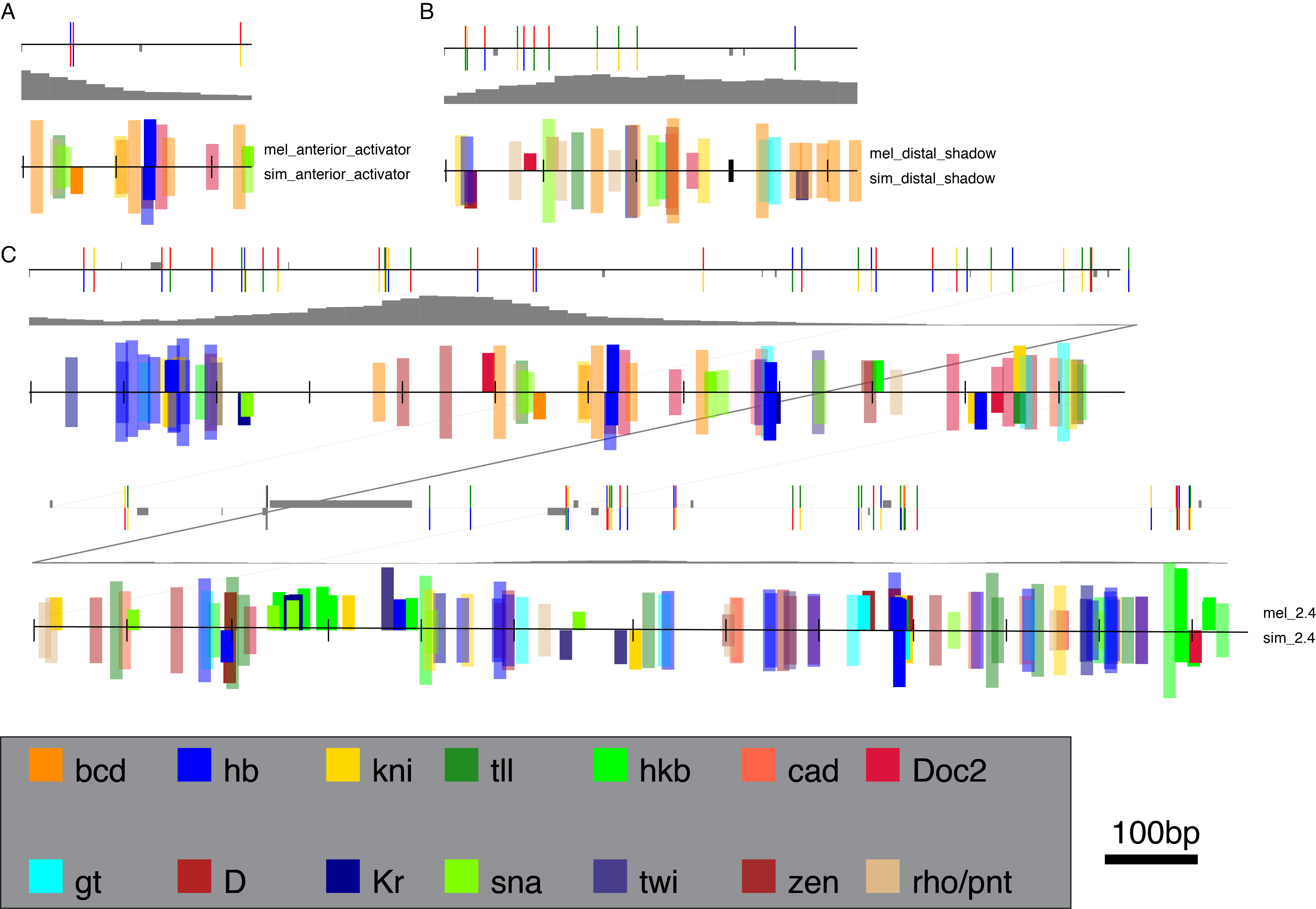

Supplement: S9 Fig — Positions of TF binding motifs in the canonical anterior CRM from [33] (A), the distal “shadow” CRM from [35] (B), and the non-minimal 2.4kb CRM construct from (of which the canonical CRM is a subset) [34], split across two lines for compactness. Within each CRM, the top line indicates the location of SNPs (colored lines) and insertions/deletions (grey bars on the side with the insertion) in a pairwise alignment of the two sequences. The middle track indicates DNase accessibility from [86]. The third track indicates the locations of FIMO motifs for a variety of TFs. TFs that have a motif with approximately equal strength (±20%) within 5bp have reduced opacity to better highlight motif changes. Bar height corresponds to FIMO score. (TIF) [file pgen.1007631.s012.tif]

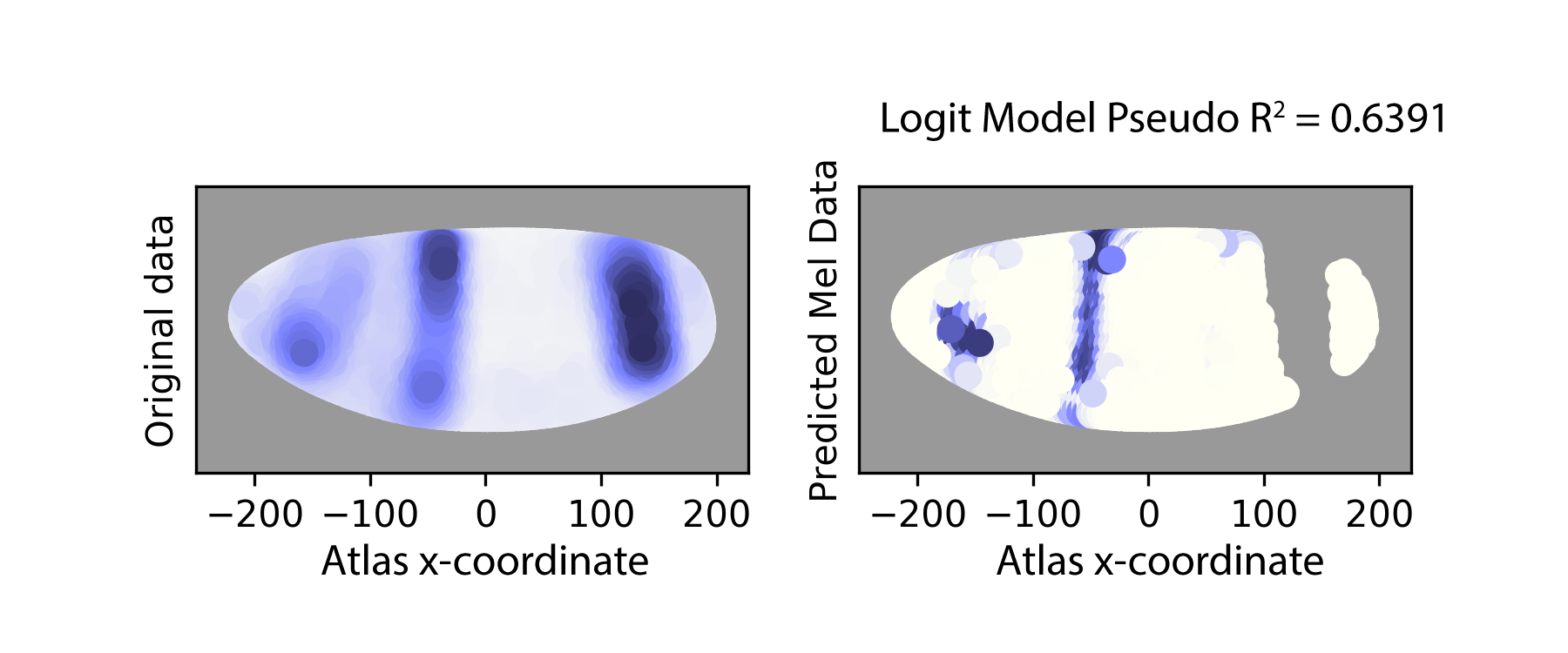

Supplement: S10 Fig — The posterior stripe of hb expression was removed prior to the fitting process. McFadden’s Pseudo R2 as reported by the statsmodels Python package. (TIF) [file pgen.1007631.s013.tif]

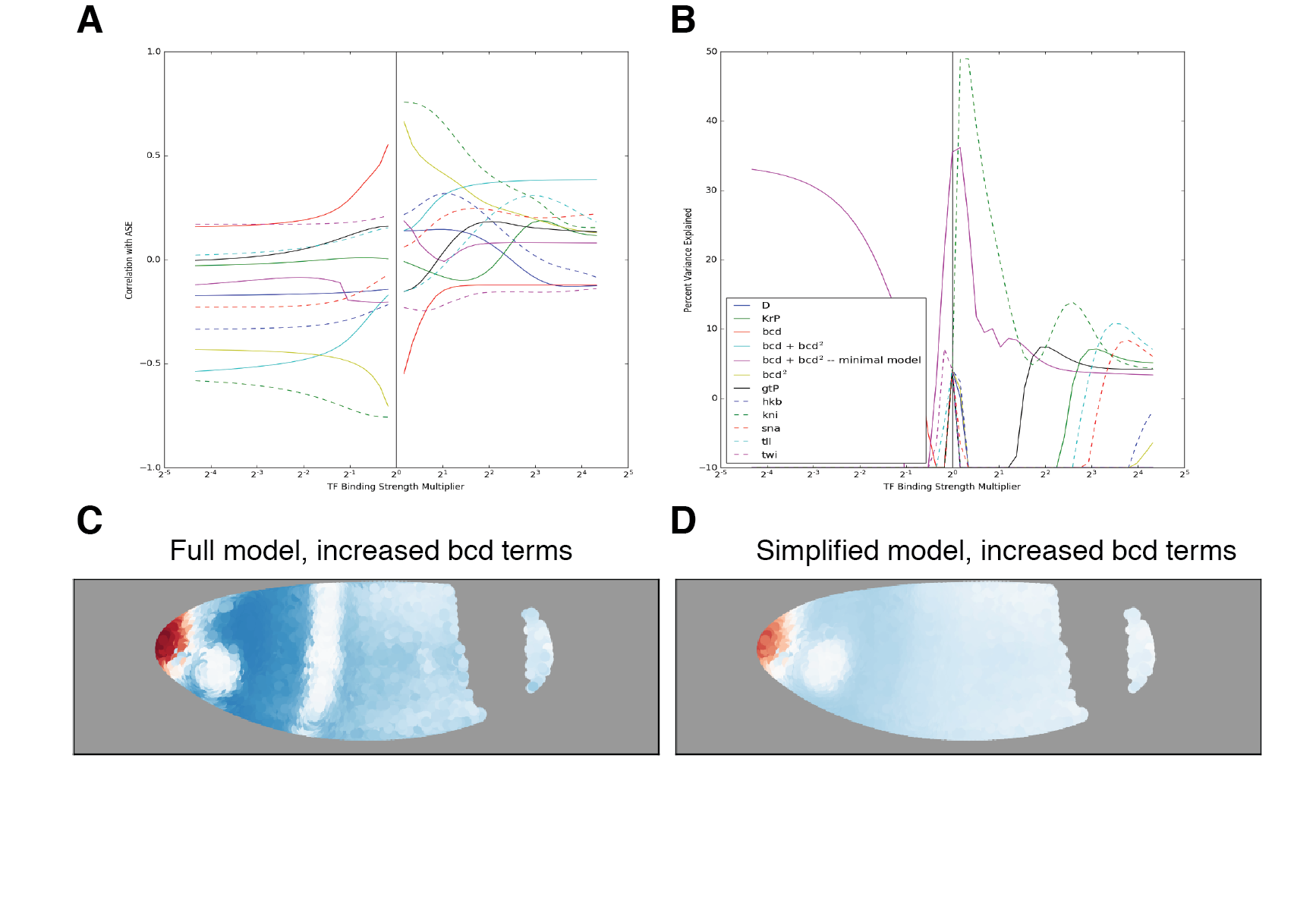

Supplement: S11 Fig — We altered each coefficient separately (with the exception of the Bicoid terms, which we also adjusted in tandem) by multiplying by a range of multipliers, then predicting ASE. Although increasing the kni term in the model had the best correlation with the real ASE, there were no Kni motif changes in the known CRMs, so we excluded it from consideration. In addition, due to the buffering effects of the other TFs in the full model, we could not find a change that, when applied to both the Bcd and Bcd2 term that explained the ASE; however, adjusting a simpler model consisting of only terms for Bcd, Bcd2, D, and twi did yield a good fit. The actual predicted ASE for these models at a given change of coefficient is qualitatively very similar (C-D). (TIF) [file pgen.1007631.s014.tif]

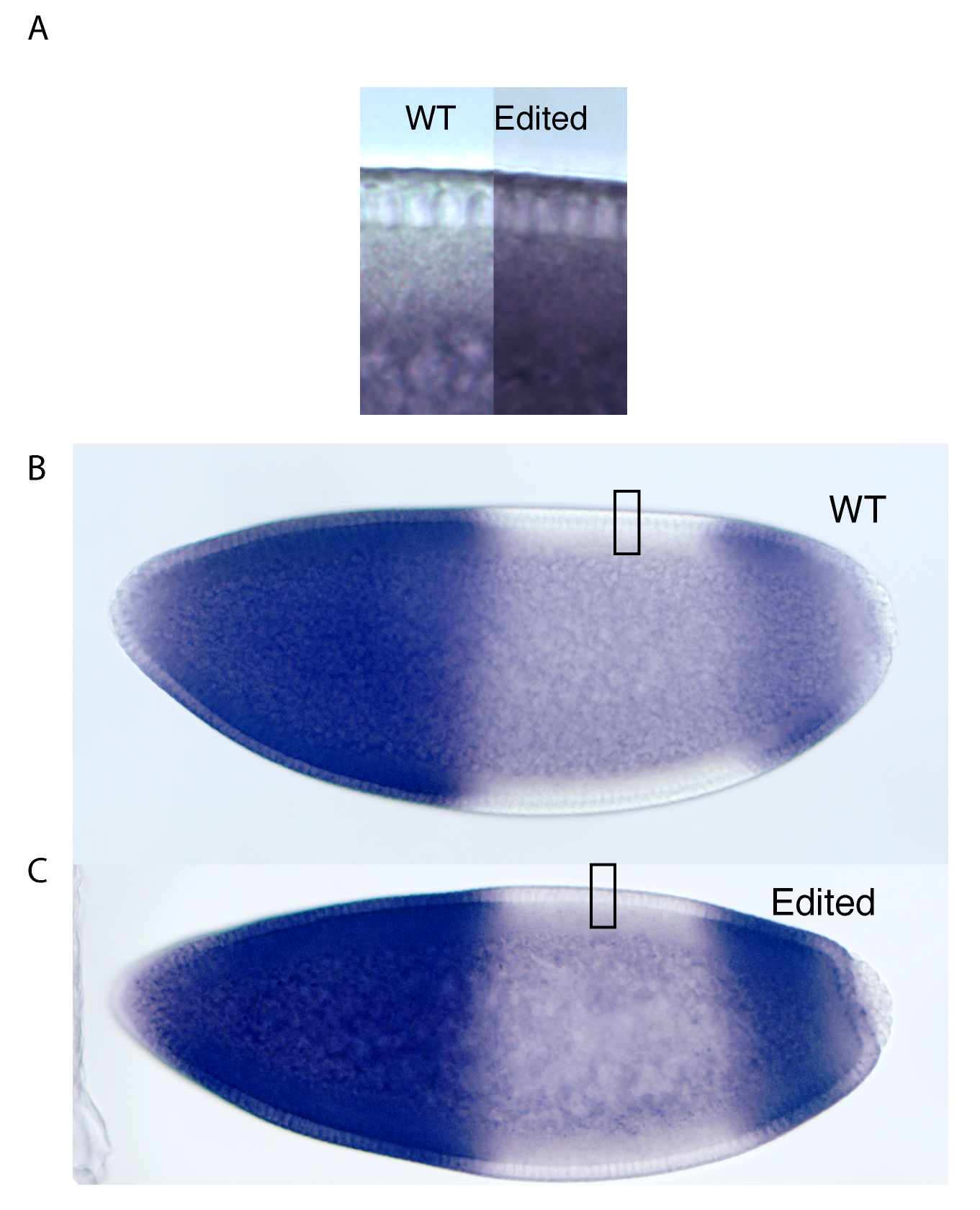

Supplement: S12 Fig — In order to find closely staged embryos, we compared the depth of the cellular membrane invagination (A) in the inter-stripe region (marked in black boxes in B and C). (TIF) [file pgen.1007631.s015.tif]

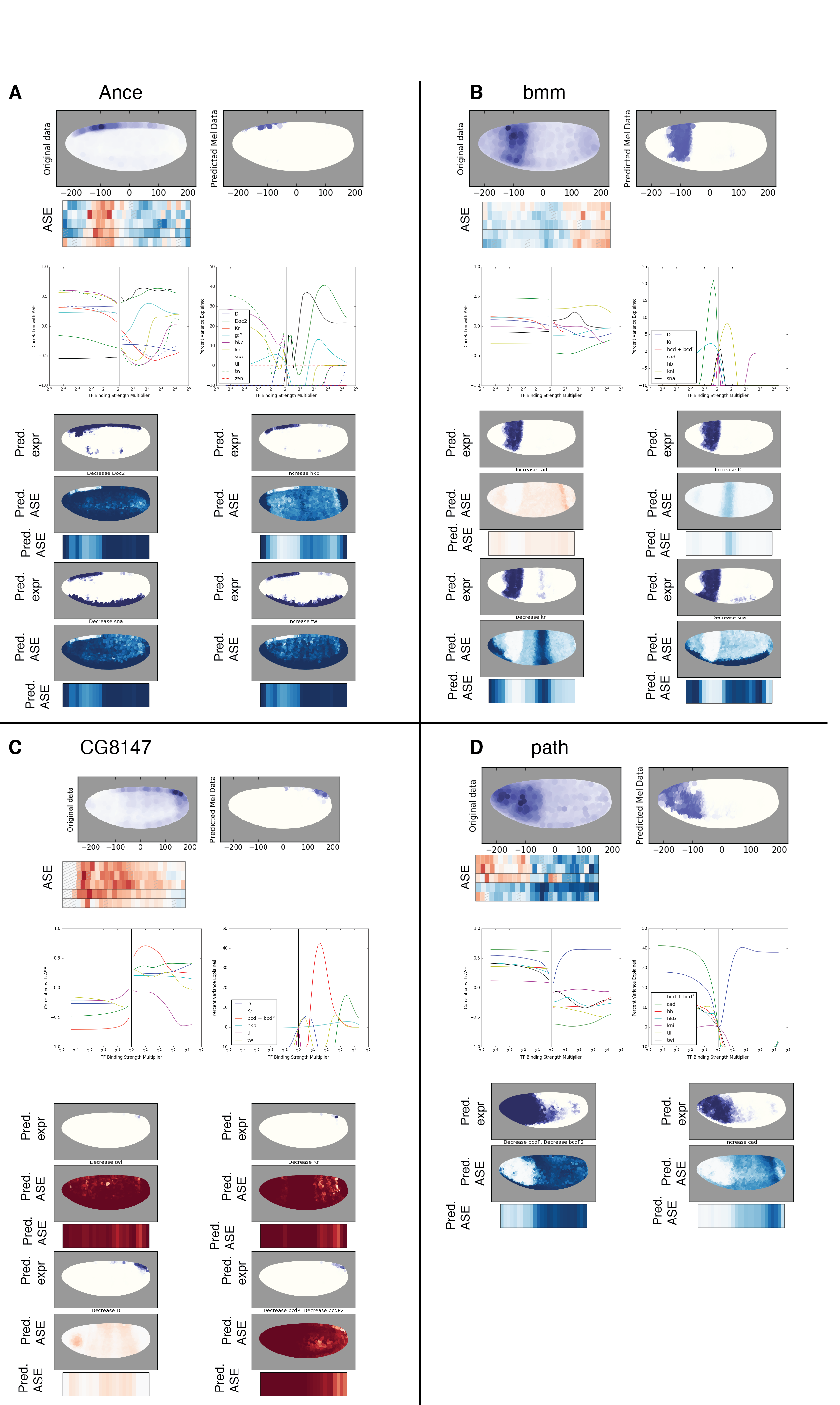

Supplement: S13 Fig — Modeling suggests plausible changes to the regulatory function that could generate the observed allele-specific expression. We fit a logistic model to the atlas expression, then adjusted each term of the model to find the coefficient that best matches the observed ASE in the slices (after setting mean ASE to match in the real and predicted data, since there may be mapping bias). The expression is then predicted in the adjusted model (purple embryo), which is also used to generate predicted ASE on a per-nucleus (red/blue embryo) and computationally sliced (heatmap) basis. Multiple TF changes can generate substantially similar sliced ASE data, while still having distinct expression patterns;in situs of the D. simulans embryos would be needed to distinguish between them. We did not attempt modeling of the pair-rule genes pxb, Bsg25A, comm2, and pxb, since other pair-rule genes have multiple, independent regulatory elements, likely complicating the modeling approach. (TIF) [file pgen.1007631.s016.tif]
